# The global, regional, and national patterns of change in the burden of edentulism, 1990–2021: an analysis of the global burden of disease study 2021 and forecast to 2041

**DOI:** 10.3389/froh.2025.1678201

**Published:** 2025-12-01

**Authors:** Xiaolan Zhong, Jian Huang, Song Zheng, Zhiyuan Wu

**Affiliations:** Department of Stomatology, Fuzhou University Affiliated Provincial Hospital, Fuzhou, China

**Keywords:** edentulism, disease burden, oral disease, prediction, trend

## Abstract

**Background:**

Edentulism is a critical global health issue. It affects physical health and quality of life, and the associated healthcare costs pose a burden on individuals and society. Therefore, we analyzed the global burden of edentulism from 1990 to 2021 and projected trends from 2021 to 2041.

**Methods:**

This study used publicly available data from the 2021 Global Burden of Disease, Injuries, and Risk Factors Study (GBD). The paper reports prevalence, incidence, and years lived with disability (YLDs) of edentulism per 100,000 population [with 95% uncertainty intervals (UI)] in 1990 vs. 2021, across all age groups (0–95 + years). It details changing trends from 1990 to 2021 by gender, age, and Socio-demographic Index (SDI). Trends were assessed using joinpoint regression. Nordpred and BAPC models projected incidence from 2021 to 2041.

**Results:**

Globally, the age-standardized incidence rate (ASIR) decreased from 328.85 (95% UI: 268.72–396.35) per 100,000 in 1990 to 305.04 (95% UI: 261.19–356.58) in 2021, with an estimated annual percentage change (EAPC) of −0.23% (95% CI: −0.33% to −0.14%), indicating a long-term decline. However, joinpoint regression revealed a recent upward trend from 2015 to 2021. The age group with the highest incidence increased by 5 years; the 75–79 group had the highest rate in 2021. The ASIR for males (284.91, 95% UI: 284.74–285.07) was lower than for females (325.06, 95% UI: 324.90–325.23) in 2021. Middle-range SDI regions tend to have high ASIR. When SDI is between 0.5 and 0.7, ASIR peaks. Incidence increased from 2015 to 2021 in both sexes. Nordpred and BAPC models predict rising incidence and case numbers from 2021 to 2041.

**Conclusions:**

Despite declining age-standardized rates, the absolute global burden of edentulism continues to rise due to population growth and aging, disproportionately affecting low-middle SDI regions, women, and older adults (≥75 years). Without intervention, incidence and burden will escalate.

## 1 Introduction

Tooth loss, a prevalent condition among adults globally, significantly affects oral health and the overall quality of life (1). It can arise from various factors including periodontitis, dental caries, and traumatic injuries. Complete tooth loss, known as edentulism, can greatly affect a person's ability to chew and digest food effectively (2). This difficulty may cause individuals to choose softer foods that are deficient in vital nutrients and fiber, potentially leading to malnutrition and associated health problems (3, 4). Edentulism is a significant health issue that not only affects oral health but also has broader implications for overall body health, including systemic diseases, nutritional status, and quality of life (5–7).

Edentulism is closely linked to socioeconomic factors (8). Studies have shown that individuals with low education and low income and resident in a rural area have a significantly higher risk of edentulism (9). Previous studies indicate that in 2021, there were an estimated 7,778,813 new cases of edentulism and 152,450,366 existing cases among the global population aged 70 and above (10). This reflects the severity of edentulism as a public health issue. The costs of treating edentulism, such as dentures, dental implants, and related medical services, can impose a significant financial burden on individuals and families. Additionally, the loss of productivity and increased care needs resulting from edentulism can have a negative impact on the socio-economic environment.

Given the substantial health and socioeconomic implications of edentulism, comprehensively assessing the disease burden of edentulism through core epidemiological metrics including prevalence, incidence, and years lived with disability (YLDs) is crucial. It would be helpful for policymakers to assess the burden of edentulism in the near future and to formulate strategies to alleviate it. Therefore, in the present study, we aimed to provide the most comprehensive and up-to-date information on the global edentulous jaw burden based on GBD 2021 and to predict its trend to 2041.

## 2 Materials and methods

### 2.1 Data source

This study utilized data from the GBD 2021 study provided by the Institute for Health Metrics and Evaluation at the University of Washington (https://vizhub.healthdata.org/gbd-results/). Detailed information on the data acquisition efforts for each iteration of GBD, including GBD 2021, can be found in the GBD capstone manuscript (11). A total of 204 countries and territories were covered by GBD 2021, which estimated the burden of 371 diseases and injuries. We chose “Global” from the database as the location, “Edentulism” for the cause, and “Incidence”, “Prevalence”, “Years Lived with Disability (YLDs)” for measures. According to the GBD 2021 Oral Disorders Collaborators, the case definition of edentulism includes any individual with no remaining permanent teeth; the edentulous state during infancy is not included (12). This case definition is consistent with clinical practice, which typically defines edentulism as the complete loss of all permanent teeth in adulthood, excluding physiological tooth loss during infancy or childhood. The disability weight used for symptomatic toothlessness leading to “great difficulty in eating meats, fruits, and vegetables” is 0.067 (0.045–0.095) as determined by the GBD disability survey. Estimates for the prevalence of edentulism were calculated using Bayesian disease meta-regression algorithm (DisModMR 2.1) (12). As would be expected for an irreversible condition, remission was fixed at zero for all ages. Incidence was inferred by DisMod-MR 2.1 through a prevalence–incidence ratio strategy (13). To make comparisons between different ages or over time easier, the age-standardized incidence rate (ASIR), age-standardized prevalence rate (ASPR) and YLDs rate were calculated. Direct age standardization was performed using the GBD 2021 world standard population. Age-standardized rates were calculated using 5-year age groups: 0–4, 5–9, 10–14, 15–19, 20–24, 25–29, 30–34, 35–39, 40–44, 45–49, 50–54, 55–59, 60–64, 65–69, 70–74, 75–79, 80–84, 85–90, 90–94 and 95+ years. The final GBD 2021 estimates used in this analysis have already undergone comprehensive quality assessment, adjustment for data gaps and biases, and imputation for missing values through the GBD's standardized modeling framework. Therefore, no additional data cleaning was required prior to our analyses.

### 2.2 Global, regional, and country-specific burden of edentulism

Using the extracted data, we described the incidence, prevalence, and YLDs of edentulism across sexes, age groups, countries, regions, and socio-demographic index (SDI). Cases were divided into 17 groups (<20, 20–24, 25–29, 30–34, 35–39, 40–44, 45–49, 50–54, 55–59, 60–64, 65–69, 70–74, 75–79, 80–84, 85–89, 90–94, and 95+ years) by age. The world is divided into 45 geographical regions. Additionally, countries and regions were categorized into five classes depending on their SDI. SDI is a composite index that considers a country's per capita income, average years of education, and fertility rate (14). It categorized 204 countries and regions into five groups: low SDI (<0.45), low-middle SDI (≥0.45 and <0.61), middle SDI (≥0.61 and <0.69), high-middle SDI (≥0.69 and <0.80), and high SDI (≥0.80). Countries with a low SDI are marked by low income, lower levels of education, and higher fertility rates, facing significant obstacles in accessing healthcare. In contrast, high SDI countries are the most developed and possess strong healthcare infrastructure. We used the SDI to explore the association between socioeconomic status and age-standardized incidence of edentulism.

To identify the key drivers of changes in the absolute burden of edentulism, we conducted a Das Gupta decomposition analysis, which partitions the total change into three additive components: (i) population size effect (the impact of changes in total population, holding age structure and age-specific rates constant); (ii) age structure effect (the impact of shifts in the age distribution, assuming constant age-specific rates); and (iii) age-specific rate effect (the impact of changes in incidence across age groups, independent of demographic changes). The analysis was performed using data stratified into four age groups: <45, 45–59, 60–79, and 80+ years. This approach helps disentangle the relative contributions of demographic changes and disease risk trends to the overall burden, thereby enhancing the policy relevance of our findings.

To account for potential non-linearity and quantify residual between-country heterogeneity, we fitted a random-effects meta-regression model using restricted maximum likelihood (REML) estimation. The association between SDI and ASR was modeled using restricted cubic splines (RCS) with 4 knots. The residual heterogeneity variance (*τ*^2^) and *I*^2^ statistic were used to quantify the magnitude of unexplained variation across countries after adjusting for SDI.

### 2.3 Trends and predictions of edentulism burden

The estimated annual percent change (EAPC) was used to assess the ASIR trends in edentulism. EAPC was calculated using linear regression models and natural logarithm data fitting, which are commonly used for ASIR trends. The formula for EAPC is EAPC = 100% × [exp(*β*) − 1], where *β* is derived from the linear regression equation y = *α* + *β*x + *ε*, y = ln(ASIR), and x represents the calendar year. The EAPC reflects the annual percentage change in the period incidence rate rather than the cumulative lifetime risk of a cohort. A negative value indicates a postponement of disease onset or a reduction in new cases. Subsequently, we analyzed the time trend of the incidence of edentulism and YLDs across different age groups from 1990 to 2021.

The joinpoint regression model was employed to assess temporal trends in the disease burden associated with edentulism. This model utilizes the least-squares method to determine the variation in illness rates, thereby circumventing the subjectivity often found in traditional trend analyses that rely on linear trends. The turning point of the changing trend was identified by calculating the sum of the squared residuals of the estimated and observed values. In our study, we used the Joinpoint Regression Program, Version 4.9.1.0 (April, 2022; Statistical Research and Applications Branch, National Cancer Institute, https://surveillance.cancer.gov/joinpoint/callable/) to analyze trends. We allowed a maximum of 5 joinpoints to balance model complexity and trend detection. The “best fit” model selection method with natural logarithm transformation (Model = ln) was used to select the optimal model. Permutation tests were conducted using the default settings in Joinpoint. APC/AAPC estimates were calculated with 95% confidence intervals.

Finally, we conducted an age-period-cohort (APC) analysis to forecast the incidence from 2021 to 2041, categorized by sex and age. APC analysis is primarily used to examine trends in the incidence and mortality of chronic diseases and to anticipate future shifts in disease burden. The analysis considered factors including age, period, and cohort. However, these three factors have a linear relationship, which can result in non-unique parameter estimates. A declining trend in the APC analysis indicates only a reduction in new-onset cases or a postponement of disease onset. The Bayesian APC (BAPC) approach addresses this by incorporating both sample data and prior information to derive unique parameter estimates, ensuring that the results are both robust and reliable (15). The Nordpred package in R software was used to analyze the incidence of edentulism in different age groups from 2021 to 2041. To verify the reliability of the predicted results, the BAPC and INLA packages in the R software were used for analysis.

All data analyses were conducted using open-source software R (version 4.3.1; R Foundation for Statistical Computing, Vienna, Austria).

## 3 Results

### 3.1 Global level

Trends in the global burden of edentulism from 1990 to 2021 are summarized in Tables 1–3. According to Table 1, globally, the ASIR decreased from 328.85 (95% UI: 268.72–396.35) per 100,000 population in 1990 to 305.04 (95% UI: 261.19–356.58) per 100,000 population in 2021, with an EAPC of−0.23% (95% CI: −0.33% to −0.14%). As Table 2 shows, the ASPR decreased from 4,515.49 (95% UI: 3,614.26–5,478.11) per 100,000 population in 1990 to 4109.24 (95% UI: 3,504.63–4,834.95) per 100,000 population in 2021, with an EAPC of 0.25% (95% CI: −0.34% to −0.16%). Table 3 indicates that the age-standardized YLDs rate also decreased from 122.64 (95% UI: 78.7–172.27) per 100,000 population in 1990 to 111.51 (95% UI: 72.25–156.83) per 100,000 population in 2021, with an EAPC of −0.24%. In most regions, we observed a declining trend in the ASIR, ASPR, and age-standardized YLDs rate. The positive EAPCs for ASIR, ASPR, and YLD rates were observed in Eastern Europe and Tropical Latin America.

**Table 1:** ASIR of edentulism between 1990 and 2021 at the global and regional level.

Group	Location	Age-Standardized Estimates (1990; 95%UI)	Age-Standardized Estimates (2021; 95%UI)	EAPC (%, 95%CI)
Global	Global	328.85 (268.72–396.35)	305.04 (261.19–356.58)	**−0.23** **(−0.33 to −0.14)**
SDI Group	High-middle SDI	351.18 (286.86–424.69)	323.61 (273.91–382.16)	**−0.30** **(−0.42 to −0.19)**
	High SDI	337.48 (274.98–410.14)	307.00 (254.76–366.37)	**−0.60** **(−0.85 to −0.35)**
	Low-middle SDI	294.08 (239.98–356.00)	268.60 (232.34–309.91)	**−0.06** **(−0.38 to 0.27)**
	Low SDI	224.39 (181.89–271.93)	208.12 (177.70–245.57)	**−0.10** **(−0.39 to 0.19)**
	Middle SDI	350.58 (286.85–420.91)	335.23 (289.54–388.97)	**−0.10** **(−0.24 to 0.04)**
Region	Andean Latin America	649.38 (565.39–729.33)	614.97 (538.26–686.78)	**−0.18** **(−0.27 to −0.10)**
	Australasia	520.71 (488.44–552.79)	377.35 (303.28–457.49)	**−0.23** **(−0.59 to 0.12)**
	Caribbean	406.30 (332.07–486.13)	382.36 (314.20–458.16)	**−0.31** **(−0.36 to −0.27)**
	Central Asia	414.80 (341.12–494.33)	378.29 (309.64–452.91)	**−0.51** **(−0.65 to −0.36)**
	Central Europe	464.33 (386.19–547.66)	416.76 (344.81–495.61)	**−0.63** **(−0.73 to −0.52)**
	Central Latin America	409.79 (338.58–486.45)	397.10 (344.34–454.79)	**−0.28** **(−0.40 to −0.16)**
	Central Sub-Saharan Africa	222.52 (179.99–267.36)	222.28 (179.81–263.96)	**−0.12** **(−0.21 to −0.03)**
	East Asia	301.22 (240.96–373.39)	287.80 (236.38–347.10)	**−0.12** **(−0.39 to 0.15)**
	Eastern Europe	414.35 (343.13–490.40)	438.30 (370.57–516.84)	0.10 (0.04 to 0.17)
	Eastern Sub-Saharan Africa	139.19 (112.19–170.76)	132.70 (107.06–162.32)	**−0.22** **(−0.26 to −0.17)**
	High-income Asia Pacific	272.07 (219.19–332.36)	214.03 (174.27–260.47)	**−1.24** **(−1.75 to −0.73)**
	High-income North America	366.95 (290.61–452.09)	323.45 (265.96–390.57)	**−0.71** **(−1.08 to −0.33)**
	North Africa and Middle East	423.05 (346.60–503.53)	396.84 (334.47–466.36)	**−0.28** **(−0.34 to −0.23)**
	Oceania	317.77 (256.75–388.42)	305.08 (245.70–372.48)	**−0.11** **(−0.14 to −0.09)**
	South Asia	271.40 (219.39–334.91)	232.28 (198.55–271.95)	**−0.04** **(−0.64 to 0.57)**
	Southeast Asia	323.19 (261.55–392.07)	292.68 (250.54–343.26)	**−0.30** **(−0.32 to −0.28)**
	Southern Latin America	351.33 (281.38–430.70)	309.44 (245.00–380.56)	**−0.42** **(−0.45 to −0.39)**
	Southern Sub-Saharan Africa	339.26 (282.59–396.45)	309.80 (254.20–364.50)	**−0.45** **(−0.73 to −0.17)**
	Tropical Latin America	595.12 (512.69–675.91)	593.82 (526.54–652.35)	0.03 (−0.11 to 0.18)
	Western Europe	329.45 (269.77–399.13)	304.07 (247.62–369.33)	**−0.53** **(−0.90 to −0.17)**
	Western Sub-Saharan Africa	168.91 (135.56–207.44)	158.91 (136.89–186.90)	**−0.52** **(−0.67 to −0.38)**

ASIR, age-standardized incidence rate; EAPC, estimated annual percentage change; UI, uncertainty interval; CI, confidence interval. Bold EAPC values indicate a declining trend.

**Table 2:** ASPR of edentulism between 1990 and 2021 at the global and regional level.

Group	Location	Age-Standardized Estimates (1990; 95%UI)	Age-Standardized Estimates (2021; 95%UI)	EAPC (%, 95%CI)
Global	Global	4,515.49 (3,614.26–5,478.11)	4,109.24 (3,504.63–4,834.95)	**−0.25** **(−0.34 to −0.16)**
SDI Group	High-middle SDI	4,794.84 (3,854.19–5,809.65)	4,235.97 (3,605.42–4,986.63)	**−0.43** **(−0.54 to −0.33)**
	High SDI	4,651.67 (3,711.25–5,719.06)	4,251.91 (3,485.21–5,101.64)	**−0.62** **(−0.90 to −0.34)**
	Low-middle SDI	3,999.29 (3,210.56–4,872.16)	3,594.21 (3,086.50–4,194.09)	0.09 (−0.33 to 0.51)
	Low SDI	3,212.02 (2,588.86–3,900.70)	2,941.12 (2,455.93–3,455.37)	0.06 (−0.33 to 0.45)
	Middle SDI	4,619.48 (3,701.34–5,578.80)	4,397.54 (3,801.94–5,110.27)	**−0.04** **(−0.20 to 0.12)**
Region	Andean Latin America	10,305.12 (8,522.86–12,212.23)	9,581.32 (8,053.57–11,152.23)	**−0.24** **(−0.34 to −0.14)**
	Australasia	9,231.77 (8,606.26–9,932.38)	5,638.90 (4,414.28–6,910.70)	**−0.49** **(−0.98 to 0.01)**
	Caribbean	5,570.10 (4,431.38–6,808.83)	5,224.25 (4,181.08–6,362.71)	**−0.32** **(−0.36 to −0.28)**
	Central Asia	6,108.96 (4,950.15–7,448.22)	5,556.27 (4,492.52–6,697.16)	**−0.56** **(−0.76 to −0.36)**
	Central Europe	6,841.11 (5,489.79–8,250.87)	6,029.14 (4,848.43–7,280.65)	**−0.71** **(−0.82 to −0.59)**
	Central Latin America	5,858.20 (4,701.14–7,062.32)	5,763.33 (4,967.78–6,682.12)	**−0.24** **(−0.36 to −0.11)**
	Central Sub-Saharan Africa	4,169.89 (3,309.59–5,059.52)	4,233.95 (3,324.14–5,126.40)	**−0.08** **(−0.19 to 0.03)**
	East Asia	3,436.84 (2,642.21–4,316.94)	3,187.75 (2,597.31–3,904.04)	**−0.15** **(−0.51 to 0.20)**
	Eastern Europe	6,023.69 (4,862.44–7,209.42)	6,610.98 (5,614.53–7,842.54)	0.21 (0.10 to 0.32)
	Eastern Sub-Saharan Africa	2,046.68 (1,620.51–2,483.53)	1,942.99 (1,546.53–2,358.69)	**−0.24** **(−0.29 to −0.19)**
	High-income Asia Pacific	3,043.47 (2,410.54–3,738.89)	2,432.19 (1,989.28–2,947.28)	**−1.46** **(−2.13 to −0.78)**
	High-income North America	5,329.66 (4,130.44–6,712.66)	5,042.56 (4,058.12–6,192.07)	**−0.55** **(−0.92 to −0.17)**
	North Africa and Middle East	6,443.03 (5,127.73–7,791.62)	5,988.86 (4,991.57–7,153.75)	**−0.33** **(−0.41 to −0.25)**
	Oceania	3,652.65 (2,867.36–4,514.92)	3,482.14 (2,726.50–4,324.48)	**−0.14** **(−0.16 to −0.11)**
	South Asia	3,454.11 (2,721.05–4,321.25)	2,876.95 (2,433.40–3,389.60)	0.36 (−0.47 to 1.21)
	Southeast Asia	4,326.47 (3,430.91–5,256.50)	3,882.66 (3,339.31–4,555.20)	**−0.29** **(−0.36 to −0.23)**
	Southern Latin America	4,795.52 (3,741.00–5,938.14)	4,162.79 (3,253.71–5,144.21)	**−0.47** **(−0.50 to −0.44)**
	Southern Sub-Saharan Africa	7,230.83 (5,742.83–8,738.83)	6,237.48 (4,968.55–7,503.89)	**−0.57** **(−0.97 to −0.17)**
	Tropical Latin America	8,753.99 (7,085.04–10,486.32)	8,896.91 (7,647.81–10,122.43)	0.11 (−0.16 to 0.38)
	Western Europe	4,453.42 (3,582.07–5,390.58)	4,076.62 (3,258.17–4,917.79)	**−0.56** **(−0.97 to −0.16)**
	Western Sub-Saharan Africa	2,556.79 (2,030.47–3,104.75)	2,382.59 (1,999.64–2,770.85)	**−0.53** **(−0.67 to −0.38)**

ASPR, age-standardized prevalence rate; EAPC, estimated annual percentage change; UI, uncertainty interval; CI, confidence interval. Bold EAPC values indicate a declining trend.

**Table 3:** Age-standardized YLDs rate of edentulism between 1990 and 2021 at the global and regional level.

Group	Location	Age-Standardized Estimates (1990; 95%UI)	Age-Standardized Estimates (2021; 95%UI)	EAPC (%, 95%CI)
Global	Global	122.64 (78.70–172.27)	111.51 (72.25–156.83)	**−0.24** **(−0.33 to −0.16)**
SDI Group	High-middle SDI	130.38 (83.59–183.55)	115.07 (75.29–161.74)	**−0.43** **(−0.53 to −0.33)**
	High SDI	126.64 (80.78–178.86)	115.68 (74.09–162.12)	**−0.62** **(−0.91 to −0.34)**
	Low-middle SDI	107.83 (70.43–150.49)	96.93 (62.73–134.70)	0.10 (−0.32 to 0.52)
	Low SDI	86.68 (56.40–120.73)	79.69 (49.99–110.90)	0.09 (−0.30 to 0.47)
	Middle SDI	125.44 (81.05–176.45)	119.23 (77.88–167.85)	**−0.04** **(−0.19 to 0.12)**
Region	Andean Latin America	284.34 (186.48–396.05)	263.53 (172.31–366.05)	**−0.25** **(−0.34 to −0.15)**
	Australasia	253.76 (170.46–349.56)	154.09 (95.94–217.43)	**−0.50** **(−1.00 to 0.00)**
	Caribbean	152.78 (98.15–215.23)	142.42 (91.88–199.27)	**−0.33** **(−0.37 to −0.29)**
	Central Asia	168.43 (109.21–233.73)	152.87 (98.94–212.94)	**−0.56** **(−0.76 to −0.36)**
	Central Europe	185.98 (120.02–257.27)	164.37 (105.75–228.35)	**−0.69** **(−0.80 to −0.58)**
	Central Latin America	159.42 (102.17–222.37)	157.10 (102.01–218.55)	**−0.23** **(−0.36 to −0.11)**
	Central Sub-Saharan Africa	114.11 (70.25–165.13)	116.69 (71.45–168.46)	**−0.05** **(−0.16 to 0.06)**
	East Asia	93.10 (59.93–133.92)	86.02 (57.29–121.31)	**−0.16** **(−0.52 to 0.20)**
	Eastern Europe	163.77 (105.95–228.16)	179.92 (113.34–249.03)	0.22 (0.11 to 0.33)
	Eastern Sub-Saharan Africa	55.70 (34.94–78.94)	53.05 (33.29–74.50)	**−0.22** **(** **−0.27 to −0.16)**
	High-income Asia Pacific	82.47 (53.67–118.84)	66.25 (44.39–94.67)	**−1.46** **(−2.13 to −0.78)**
	High-income North America	145.26 (91.09–206.45)	136.66 (85.76–191.23)	**−0.56** **(−0.93 to −0.20)**
	North Africa and Middle East	176.70 (110.78–246.97)	163.15 (104.76–227.97)	**−0.35** **(−0.43 to −0.27)**
	Oceania	97.93 (62.78–140.35)	93.15 (60.52–133.68)	**−0.14** **(−0.16 to −0.11)**
	South Asia	92.07 (59.99–130.72)	76.76 (49.39–108.61)	0.41 (−0.44 to 1.26)
	Southeast Asia	117.63 (74.82–165.93)	105.68 (68.69–149.21)	**−0.28** **(−0.35 to −0.21)**
	Southern Latin America	130.92 (82.40–189.13)	113.11 (71.66–164.42)	**−0.48** **(−0.51 to −0.45)**
	Southern Sub-Saharan Africa	200.75 (123.38–293.49)	171.19 (106.68–248.41)	**−0.60** **(−1.00 to −0.20)**
	Tropical Latin America	236.43 (154.89–335.69)	240.61 (156.77–330.08)	0.12 (−0.16 to 0.40)
	Western Europe	121.31 (77.14–174.66)	111.13 (71.38–158.58)	**−0.56** **(−0.97 to −0.15)**
	Western Sub-Saharan Africa	69.78 (43.77–98.15)	65.31 (40.78–90.45)	**−0.51** **(−0.65 to −0.36)**

YLDs, Years lived with disability; EAPC, estimated annual percentage change; UI, uncertainty interval; CI, confidence interval. Bold EAPC values indicate a declining trend.

**Figure 1:**
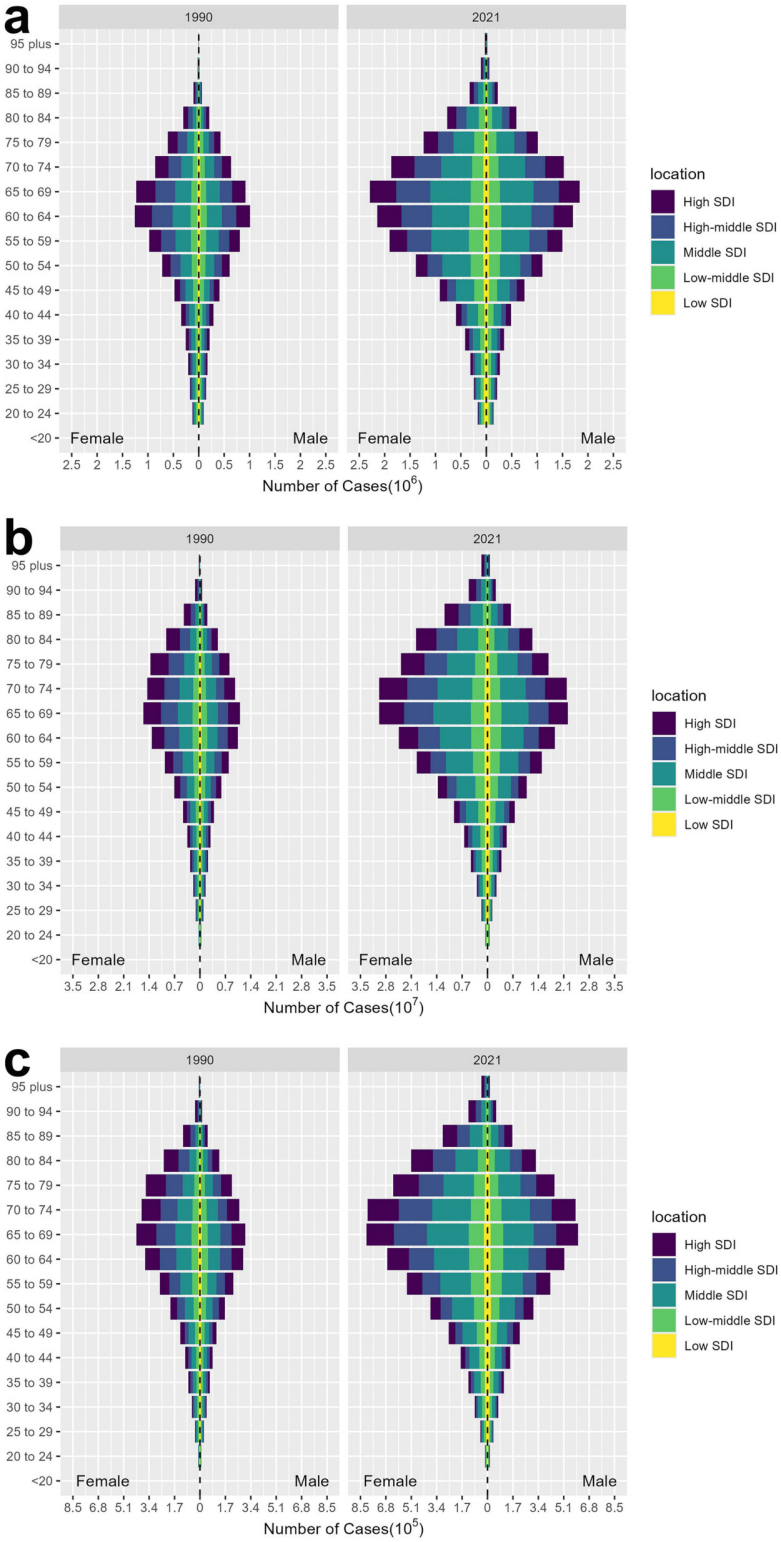
Number of incidence **(a)**, prevalence **(b)** and YLDs **(c)** by sex, age and SDI for 1990 and 2021. SDI: Sociodemographic Index; YLDs: Years Lived with Disability.

The burden of edentulism in 1990 and 2021 is summarized in Figure 1. From 1990 to 2021, the number of incident cases, prevalent cases, and YLDs has increased. As depicted in Figure 1a, in 1990, incident cases increased across age groups, peaking at the age group of 60–64 years, and began to decline in the 65–69 age group. The greatest burden of new edentulism cases occurred among adults aged 60–64 years in the middle-SDI region, with approximately 297,936 incident cases in males and 348,620 in females. In 2021, the number of new edentulism cases increased with age and peaked in the 65–69 group—five years older than the peak age group in 1990. The 65–69 years age group recorded the highest number of new edentulism cases: approximately 658,569 among males and 790,880 among females in the middle-SDI region. The prevalent cases of edentulism in 1990 and 2021 are summarized in Figure 1b. In 1990, the number of prevalent cases rose with increasing age, reaching a peak in the 65–69 years age group, followed by a decline in the 70–74 years and older age groups. The highest number of prevalent cases was observed in the 65–69 years age group, with approximately 3,031,453.68 males and 4,029,031.60 females affected in the middle-SDI region. By 2021, the burden of edentulism peaked in both the 65–69 and 70–74 years age groups, with little difference between them. In the middle-SDI region, there were approximately 7,503,557.6 males and 10,305,261.9 females aged 65–69 with edentulism, compared to about 7,056,911.3 males and 9,589,313.2 females in the 70–74 age group. The YLDs of edentulism in 1990 and 2021 are summarized in Figure 1c. The number of YLDs has increased among people younger than 69 years and decreased among people aged 70 years and above in both 1990 and 2021. The 65–69 age group had the highest YLDs in both years. In 1990, the YLDs for males in the middle SDI region was approximately 83,310 and for females in the middle SDI region was approximately 109,939. In 2021, the YLDs for males in the middle SDI region was approximately 205,829 and for females in the middle SDI region was approximately 280,465.

The Das Gupta decomposition revealed that the increase in the absolute burden of edentulism was primarily driven by demographic changes (Supplementary Table S1). Population growth accounted for 62.19% (+7.65 billion cases) of the total increase, while shifts in age structure (i.e., population aging) contributed an additional 50.63% (+6.23 billion cases). These two factors combined explain over 110% of the observed rise. In contrast, changes in age-specific rates had a mitigating effect, reducing the burden by 12.38% (−1.52 billion cases), likely due to improvements in oral health and dental care. The residual was negligible (−0.44%), indicating good model fit and additive consistency.

### 3.2 National level

**Figure 2:**
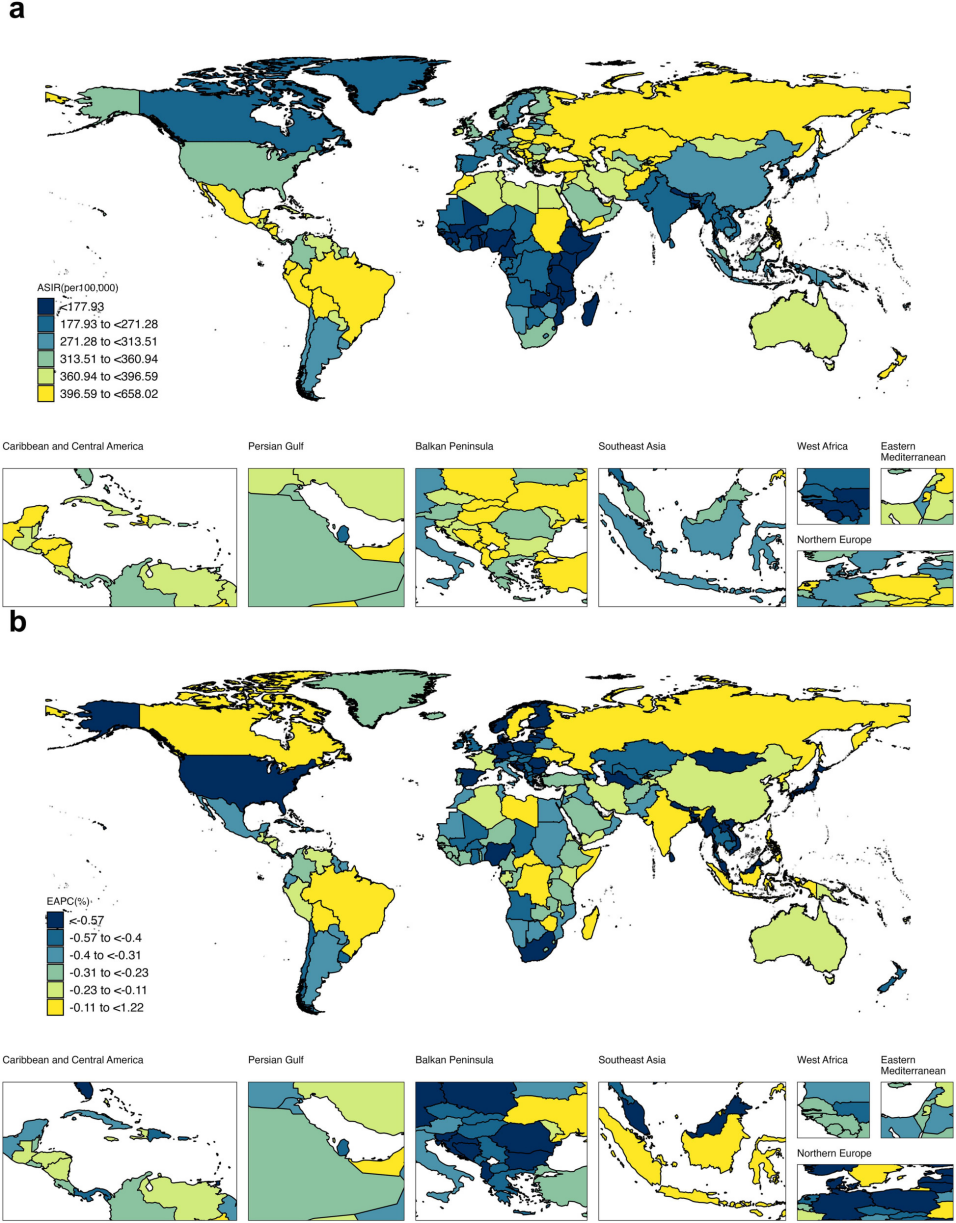
Global disease burden of edentulism for both sexes in 204 countries and territories. **(a)** ASIR of edentulism in 2021. **(b)** EAPC for edentulism from 1990 to 2021. ASIR: age-standardized incidence rate; EAPC: estimated annual percentage change.

The global burden of edentulism at the national level is summarized in Figure 2 and Supplementary Table S2. The ASIR in 2021 varied from 106.34 (95% UI: 83.92 to 133.25) to 658.02 (95% UI: 569.01 to 736.10) per 100,000 population (Figure 2a). The largest ASIR was observed in the Plurinational State of Bolivia (658.02 per 100,000 population; 95% UI: 569.01 to 736.10), followed by Peru (630.02 per 100,000 population; 95% UI: 542.75 to 710.04), Brazil (598.99 per 100,000 population, 95% UI: 529.47 to 657.95), Ecuador (555.66 per 100,000 population; 95% UI: 467.34–641.75), and Poland (477.81 per 100,000 population95% UI: 396.10–562.95). The top 10 countries with the largest ASIR in 2021 are further presented in Supplementary Figure S1. To obtain a picture of the ASIR trends from 1990 to 2021, the EAPCs were calculated and summarized in Figure 2b. Palau had the highest ASIR increase rate (EAPC: 1.22%, 95% CI: 1.00% to 1.45%), followed by Tuvalu (EAPC: 1.19%, 95% CI: 0.95% to 1.42%), Nauru (1.08%, 95% CI: 0.91% to 1.25%), Sweden (EAPC: 0.54%, 95% CI: 0.12% to 0.96%), and the Democratic People's Republic of Korea (EAPC: 0.46%, 95% CI: 0.44% to 0.48%). In some countries, the ASIR decreased from 1990 to 2021, such as Finland (EAPC: −1.52%; 95% CI: −2.03% to −1.01%), Japan (EAPC: −1.42%; 95% CI: −2.12% to −0.72%), and Equatorial Guinea (EAPC: −1.22%; 95% CI: −1.39% to −1.05%). Specifically, the changes in ASIR from 1990 to 2021 for the 5 countries with the highest EAPC and the 5 countries with the lowest EAPC are illustrated in Supplementary Figure S2.

### 3.3 The association between the ASIR and SDI

**Figure 3:**
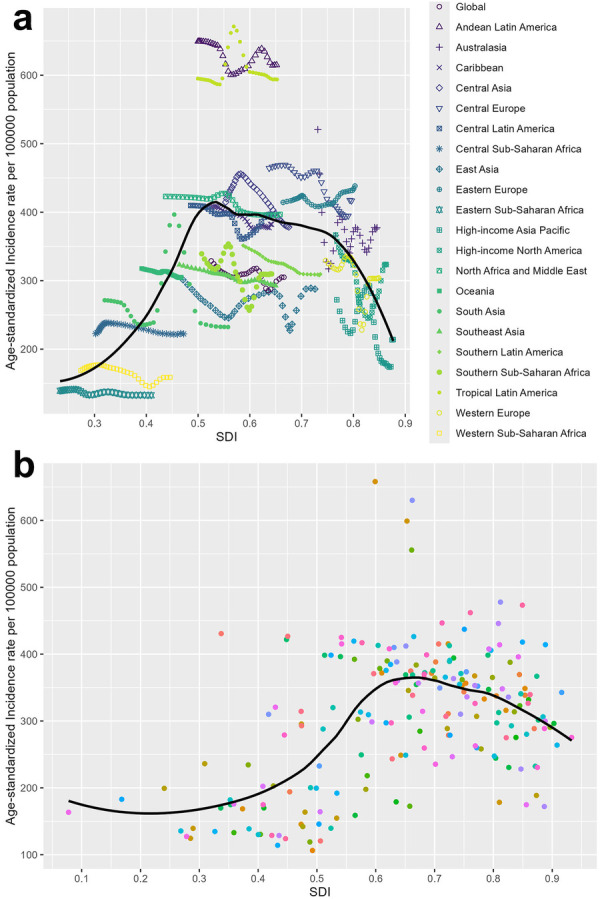
Relationship between SDI and ASIR at both the regional levels **(a)** and national levels **(b)** SDI: sociodemographic index; ASIR: age-standardized incidence rate.

We further investigated the relationship between the SDI and ASIR at both regional and national levels, with the findings presented in Figure 3. As shown in Figure 3a, regions with a middle-range SDI tended to have a relatively high ASIR. Specifically, when a region's SDI falls between 0.5 and 0.7, the ASIR reaches its peak. For instance, Andean Latin America and Tropical Latin America, both with SDIs between 0.5 and 0.7, exhibit the highest ASIR among all regions. The disparity in the ASIR can be significant in different regions with a similar SDI. This pattern is also evident at the national level, where countries with an SDI between 0.6 and 0.7 show a similar trend (Figure 3b).

We used restricted cubic splines to model the non-linear association between SDI and ASIR. A significant inverted U-shaped relationship was identified, with peak burden at SDI = 0.75 (*p* < 0.001 for non-linearity; *R*^2^ = 0.49) (Supplementary Figure S3, Supplementary Table S3). Random-effects meta-regression revealed substantial residual heterogeneity (*I*^2^ = 90.22%), but confirmed a significant non-linear trend (QM = 107.54, df = 3, *p* < 0.001) (Supplementary Table S4).

### 3.4 Trend of different ages

**Figure 4:**
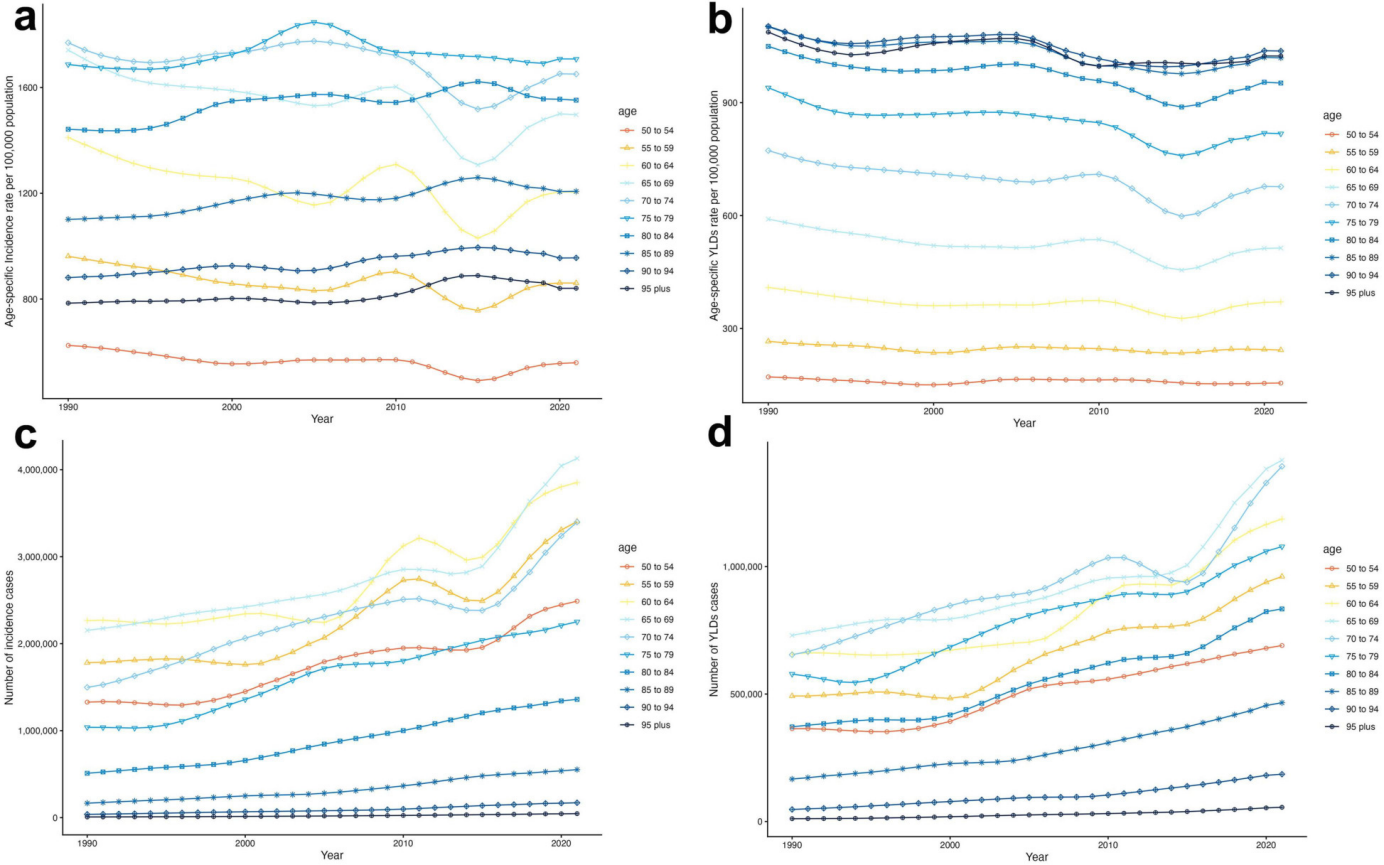
Time trends in the incidence rate **(a)** and years lived with disability (YLDs) **(b)**, as well as the number of incident cases **(c)** and YLDs **(d)** due to edentulism among individuals aged 50 years and older, from 1990 to 2021.

The time trends of the incidence and YLDs of edentulism across different age groups are summarized in Figure 4. As Figure 4a shows, over the past 32 years, the incidence rate for those under 50 years has remained relatively unchanged (Supplementary Figure S4). The incidence rate for those over 75 years of age has increased, while the incidence rate for those aged 50–74 has decreased. The 75–79 years age group had the highest incidence rate per 100,000 population. Conversely, as illustrated in Figure 4b, the YLDs rate consistently declined each year, with higher YLDs rates observed in older age groups.

### 3.5 Joinpoint regression analysis

**Figure 5:**
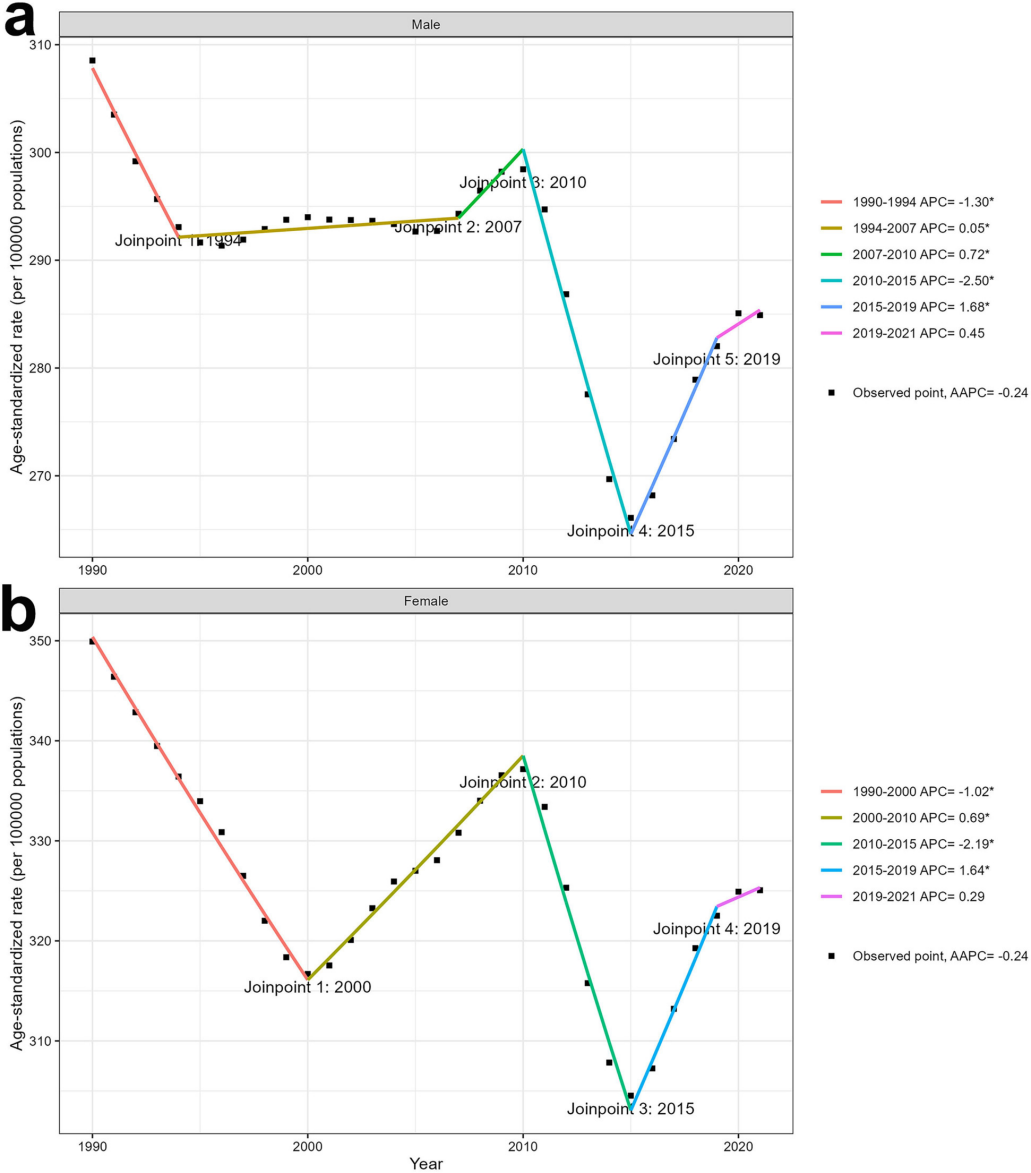
Joinpoint regression analysis of sex-specific ASIR for edentulism from 1990 to 2021. **(a)** ASIR for males. **(b)** ASIR for females. ASIR: age-standardized incidence rate; APC: annual percent change. *Indicates that the Annual Percent Change is significantly different from zero at the alpha = 0.05 level.

Joinpoint regression analyses of the ASIR for edentulism from 1990 to 2021 are shown in Figure 5 and Supplementary Table S5. From 1990 to 2010, the trend in incidence differed between males (Figure 5a) and females (Figure 5b). For females, the decrease in the ASIR was more pronounced from 1990 to 2000, whereas the change in the trend for males during the same period was not as noticeable. The disease incidence trend significantly decreased from 2010 to 2015 and appeared to increase from 2015 to 2021 in both male and female populations. However, this recent increase should be interpreted with caution due to potential influences from GBD modeling updates and data availability.

### 3.6 The predicted incident rate and case

**Figure 6:**
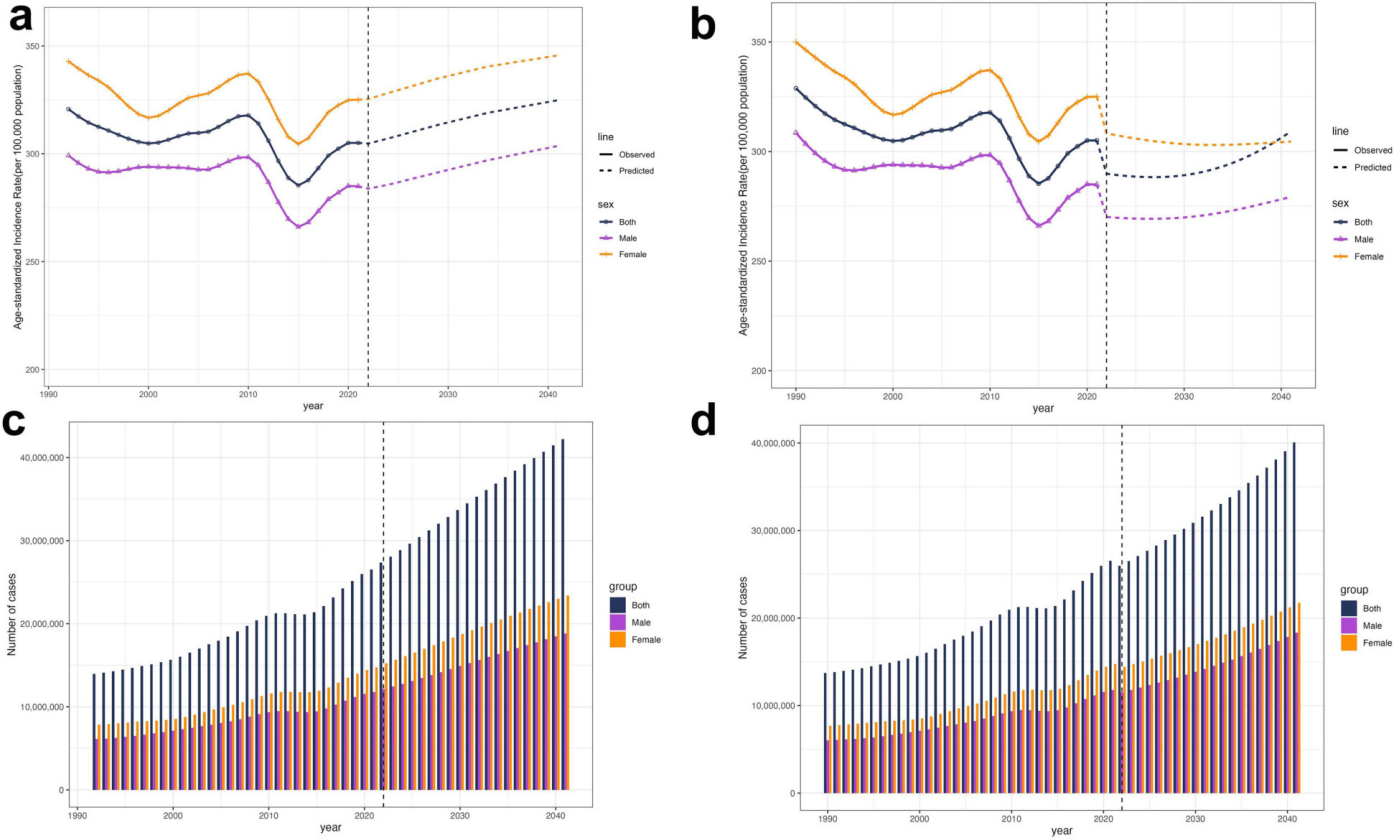
Statistics and predictions of incidence in all sex groups: **(a)** ASIR according to Nordpred model. **(b)** ASIR according to BAPC model. **(c)** Incidence number according to Nordpred model. **(d)** Incidence number according to BAPC model. ASIR: age-standardized incidence rate.

**Figure 7:**
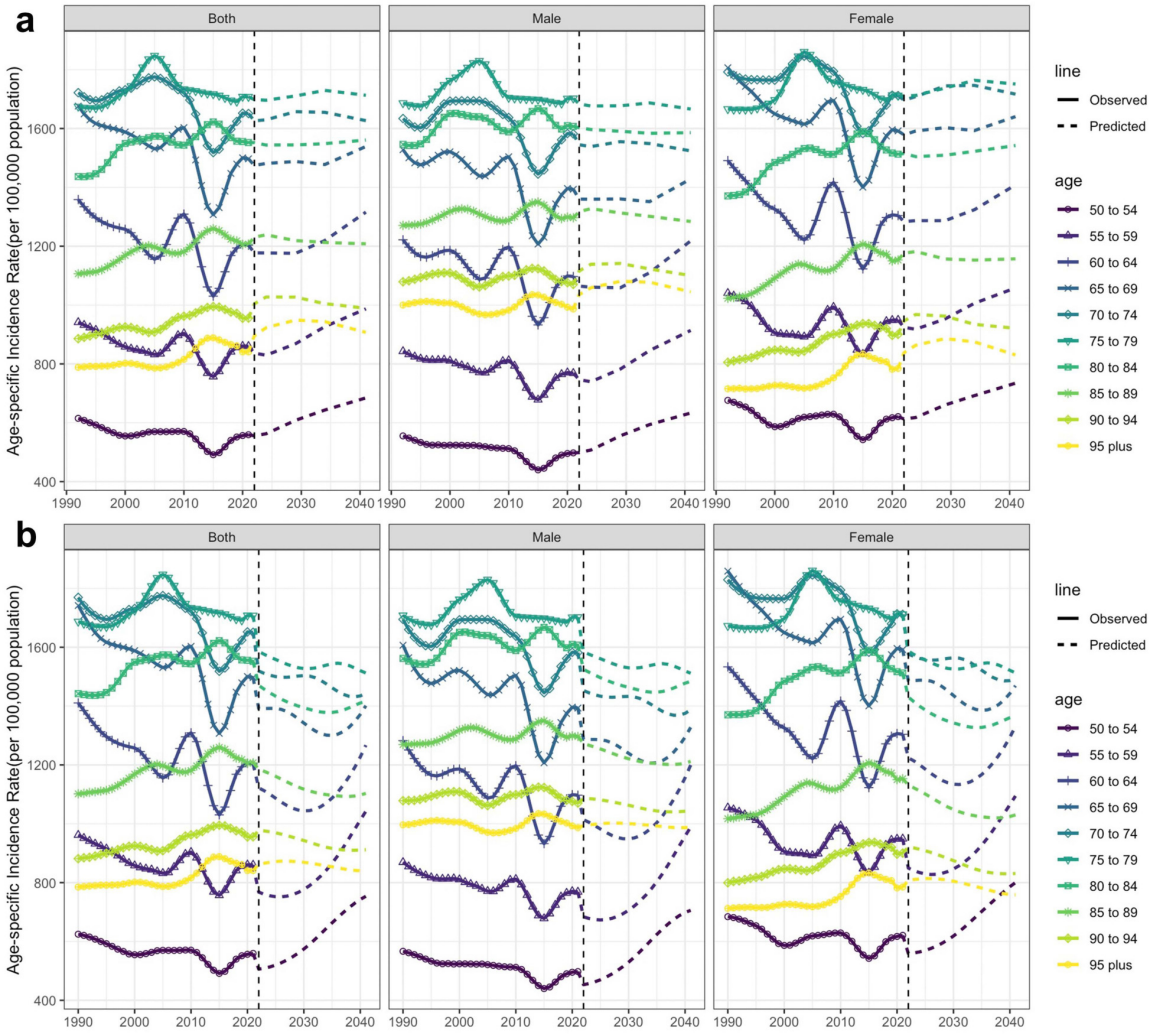
Statistics and predictions of incidence in all age groups: **(a)** ASIR according to the nordpred model. **(b)** The ASIR according to the BAPC model. ASIR: age-standardized incidence rate.

The APC results are shown in Figure 6. Generally, the ASIR will increase in the following years. According to the Nordpred model analysis (Figure 6a), the ASIR was 328.85 per 100,000 population in 1990, then fluctuated and dropped to 305.04 in 2021, and then rose to 324.85 in 2041. The prediction results of the BAPC model (Figure 6b) showed a similar trend change, but the magnitude of the change was not as significant as that of the Nordpred model. The ASIR was 328.85 per 100,000 population in 1991, then fluctuated and dropped to 305.04 in 2021, and then rose to 309.24 in 2041. Nordpred model analysis showed that ASIR in females increased from 325.06 in 2021 to 345.62 in 2041, and ASIR in males increased from 284.91 in 2021 to 303.65 in 2041. However, the prediction results of the BAPC model for different sexes are the opposite. The ASIR for females decreased from 325.06 in 2021 to 304.59 in 2041, while the ASIR for males slightly decreased from 284.91 in 2021 to 279.24 in 2041. To resolve this discrepancy, we conducted additional out-of-sample validation (training set: 1990–2011; validation set: 2012–2021). Results showed the BAPC model had significantly lower errors—with global Mean Squared Error (MSE) of 170.19 (vs. 807.64 for Nordpred) and Mean Absolute Percentage Error (MAPE) of 3.82% (vs. 9.34% for Nordpred)—confirming its higher prediction accuracy and more reliable results (Supplementary Table S6). In terms of number, the incidence predicted by both models increased (Figures 6c,d). By analyzing the incidence rates across different age groups, our study found that in the Nordpred model (Figure 7a), the incidence rate of edentulism for individuals aged 50–64 gradually increases after 2021. In the BAPC model (Figure 7b), the incidence rate for this age group initially decreased after 2021 and then increased; however, the final result was an overall increase. For other ages, there were differences in the predicted incidence rates between the two models. The Nordpred model suggests a slight increase, whereas the BAPC model shows a slight decrease in some parts. The age group of 75–79 remains the one had the highest incidence rate.

## 4 Discussion

Despite progress in curative and preventive dentistry, edentulism remains a significant global challenge for healthcare providers (16). This condition is often associated with various comorbidities that can significantly affect an individual (17, 18). Although the rate of edentulism has declined, the number of edentulous individuals is increasing (19). Our study reached a similar conclusion. From 1990 to 2021, age-standardized rates of edentulism incidence, prevalence, and YLDs per 100,000 population declined globally. However, the absolute number of incident cases, prevalent cases, and YLDs increased over this period. In terms of age, the peak age group of incident cases in 2021 was five years older than the peak age group observed in 1990. The observed shift in peak age may be attributed to improved preventive care, widespread use of fluoridated toothpaste, and greater oral health awareness (20, 21). The shift toward an older peak age group may also be influenced by extended life expectancy and population aging, allowing more individuals to survive into ages where complete tooth loss becomes more prevalent—a phenomenon consistent with survival bias (22). Our findings support this hypothesis: the Das Gupta decomposition revealed that population growth and aging together accounted for over 110% of the increase in absolute burden, with population size and age structure effects contributing 62.19% and 50.63%, respectively. This indicates that demographic shifts—particularly the expanding older population due to increased life expectancy—are key drivers of rising edentulism burden, independent of changes in disease risk.

The burden of edentulism varies by country and region. In 2021, the rates and numbers of incidence, prevalence, and YLDs were the highest in the middle SDI regions across different age groups. The five countries with the highest ASIR also included four countries from the low-middle or middle SDI regions. Similarly, among the five countries with the highest EAPC, three were from the low-middle or middle SDI regions. These results illustrate the effect of socioeconomic conditions on the risk of edentulism. The inverted U-shaped association between SDI and ASIR, with peak burden at SDI = 0.749, suggests that countries undergoing socioeconomic transition may face the greatest risk—where rising sugar consumption and aging populations coincide with limited access to preventive and restorative dental care. Adverse demographic and socioeconomic conditions were associated with the highest percentage of edentulous individuals. The primary factors influencing edentulism among the 'older adult were age, educational attainment, and socioeconomic status (23). Due to differences in socioeconomic conditions and healthcare policies across various countries or regions (24–27), the burden of edentulism also varies. The substantial residual heterogeneity across countries (*I*^2^ = 90.22%) further indicates that factors beyond national development level—such as oral health promotion, dental workforce distribution, and prosthodontic service coverage under universal health coverage (UHC)—may play critical roles in shaping edentulism burden. For instance, Japan's universal health insurance system has significantly increased dental service utilization by reducing out-of-pocket expenses, thereby decreasing the proportion of edentulism among adults aged 65–74 years to 6.9% in 2011, which is much lower than the average of 13.3% reported in 15 European countries in 2013 (28). A 20% sugar-sweetened beverages tax in Australia would lead to a reduction in decayed, missing, and filled teeth by 3.9 million units over 10 years, potentially averting hundreds of thousands of future edentulism cases over decades (29). The positive EAPCs for ASIR, ASPR, and YLD rates in Eastern European and Tropical Latin America are also associated with insufficient preventive measures for adults and the 'older adult, high rates of tooth extraction, high costs of dental care, high smoking rates, and high sugar intake (30, 31). It is necessary to adopt policies tailored to specific situations to improve the burden of edentulism.

From the trend of edentulism across different age groups, the incidence rate has decreased in the 50–74 age group, while it has increased in those aged 75 years and above. During this process, the age group of 75–79 gradually surpassed the 70–74 age group, becoming the one with the highest incidence rate. This shift suggests that the onset of edentulism is being delayed, reflecting improved oral health outcomes in later life. The decline in the YLDs rate suggests that the impact of edentulism on the quality of life of the 'older adult population is diminishing. This demonstrates that many effective measures have been implemented in recent years, and these measures should continue to be pursued in future years.

Based on Joinpoint regression analyses of the ASIR for edentulism from 1990 to 2021, the ASIR for both males and females exhibited a fluctuating decline. Two inflection points were identified, with ASIR increasing after 2000 and 2015, and continuing to rise in 2021. Overall, the ASIR for males was lower than that for females. The differences in ASIR between genders may be related to gender disparities in education and income (32, 33). The increase in ASIR after 2000 may be related to the rise in patient consultation rates, as consultation rates for many diseases have increased since 2000 (34, 35). Since 2015, the pace of aging has accelerated, whereas the growth rate of the working-age population has slowed. The growth rate of the population aged 65 and over is five times that of the working-age population (36). This may explain the gradual increase in the incidence of edentulism after 2015. However, this recent increase should be interpreted cautiously, as it may also reflect updates in the GBD modeling framework, such as expanded data inputs or revised estimation methods. Nevertheless, given the ongoing and projected acceleration of population aging—a key risk factor for edentulism—the burden may continue to rise in the future (37).

In the projected outcomes for the next 20 years, both the ASIR and the incidence number of edentulism are expected to increase globally. Therefore, alleviating the global burden of edentulism is an important international issue. The ASIR for males is lower than that for females. The 75–79 age group still exhibited the highest incidence rate. The incidence of edentulism among patients aged 50–64 should not be overlooked, as the incidence rate is anticipated to grow the fastest over the next two decades. Notably, this projection's assumption of no major innovations or policy interventions may result in an overly pessimistic interpretation of rising global edentulism ASIR trends. Evidence suggests several factors could alter this trajectory. Technological advances in areas such as regenerative dentistry and AI-enhanced early diagnosis could specifically target the prevention of complete tooth loss (38, 39). Similarly, expanded policy actions including integration of essential oral care into UHC and strengthened community prevention programs could disrupt key disease pathways to edentulism (40). For example, in the United States, states that expanded Medicaid coverage saw an 11.4 percentage point increase in dental visits, a 16.8 percentage point decrease in the prevalence of untreated dental caries, and an 8.7 percentage point decrease in the prevalence of functional dental problems, compared to states that did not expand coverage (41). Finally, improved oral health literacy might promote earlier treatment-seeking behavior to preserve natural dentition, thereby reducing progression to edentulism (42). While realizing these factors remains uncertain, their consideration provides critical balance to future outlooks and underscores the need for proactive interventions to mitigate these projected trends.

This study had several limitations. First, data on potential region-level moderators—such as sugar consumption, smoking prevalence, and dental workforce density—were not consistently available across all 204 countries, limiting our ability to adjust for or explore their modifying effects on the SDI–ASIR relationship. Second, the primary data sources used in GBD2021 vary in quality and methodology across low- and middle-income countries (LMICs), introducing potential heterogeneity and bias due to differences in diagnostic criteria, survey coverage, and reporting practices. Third, despite adjusting for key risk factors, residual confounding by unmeasured social, behavioral, and environmental determinants of oral health—such as access to care, oral hygiene practices, and socioeconomic inequalities—may persist, potentially influencing observed trends. Fourth, due to the aggregated nature of the exposure data, we were unable to disentangle and apportion the causal contributions of untreated caries vs. periodontitis to the burden of edentulism, which may have distinct etiological pathways and public health implications. Fifth, we could not quantitatively assess the extent to which the increase in the global burden of edentulism could be attributed to population growth and aging, as GBD estimates are already age-standardized and population-adjusted. Sixth, our projection models (*N*ordpred and BAPC) lacked external validation against independent national datasets, such as population-based oral health surveys. Finally, our forecasts are subject to structural uncertainty inherent in modeling assumptions, including the stability of current trends and the absence of unanticipated policy or technological interventions. Future studies should consider validating model outputs using datasets like the Behavioral Risk Factor Surveillance System (BRFSS) or national oral health examination surveys to assess predictive accuracy and improve model reliability.

## 5 Conclusions

Despite the declining trend in the ASIR and ASPR of edentulism globally, its absolute burden continues to rise, driven by population growth and aging. The distribution of the disease shows significant inequality: regions with low to middle SDI bear the heaviest burden, and women and older age groups (75 years and above) are at higher risk. Over the next two decades, without significant interventions, the incidence and standardized burden are expected to continue increasing, with particular attention needed for the accelerating rate among the 50–64 age group. However, by integrating strategies such as regenerative dental technologies, universal oral health care coverage, and health literacy improvement, this trend can potentially be reversed. Countries urgently need to develop targeted prevention and control policies addressing socioeconomic disparities to mitigate this preventable global health challenge.

## Data Availability

The original contributions presented in the study are included in the article/Supplementary Material, further inquiries can be directed to the corresponding author.
