# Supplementary material for: The global, regional, and national patterns of change in the burden of edentulism, 1990–2021: an analysis of the global burden of disease study 2021 and forecast to 2041
Source: Front Oral Health. 2025 Dec 1;6:1678201. doi: 10.3389/froh.2025.1678201 (PMC12702965; doi:10.3389/froh.2025.1678201)
Supplement: Supplementary file 1 [file Table1.docx]

**Table S1 Drivers of changes in absolute burden based on a Das Gupta decomposition analysis**

| Effect_Type | Value | Percentage (%) |
| --- | --- | --- |
| Population size effect | 7,647,981,103 | 62.19 |
| Age structure effect | 6,226,340,960 | 50.63 |
| Age-specific rate effect | -1,522,483,343 | -12.38 |
| Total change (observed) | 12,298,262,110 | 100.00 |
| Total decomposition | 12,351,838,721 | 100.44 |
| Residual | -53,576,611 | -0.44 |

**Table S2 Age-standardized incidence rate (ASIR) and estimated annual percentage changes (EAPC) of edentulism**

| location | ASIR(95% UI) | EAPC(95% CI) |
| --- | --- | --- |
| Bolivia (Plurinational State of) | 658.02(569.01 to 736.10) | 0.06(0.02 to 0.11) |
| Peru | 630.02(542.75 to 710.04) | -0.17(-0.22 to -0.13) |
| Brazil | 598.99(529.47 to 657.95) | 0.04(-0.11 to 0.19) |
| Ecuador | 555.66(467.34 to 641.75) | -0.38(-0.69 to -0.07) |
| Poland | 477.81(396.10 to 562.95) | -0.71(-1.03 to -0.40) |
| United Arab Emirates | 473.21(388.75 to 556.17) | 0.10(0.08 to 0.13) |
| Ukraine | 461.97(382.94 to 551.35) | 0.15(0.12 to 0.19) |
| Turkey | 446.61(366.68 to 532.15) | -0.27(-0.54 to -0.00) |
| Russian Federation | 445.86(372.34 to 532.11) | 0.15(0.07 to 0.22) |
| North Macedonia | 437.19(359.18 to 534.99) | -0.44(-0.52 to -0.37) |
| Afghanistan | 430.68(352.76 to 512.98) | -0.24(-0.31 to -0.17) |
| Yemen | 426.67(346.36 to 509.87) | -0.19(-0.25 to -0.13) |
| Mexico | 426.25(352.23 to 515.96) | -0.34(-0.57 to -0.11) |
| Tajikistan | 424.98(348.77 to 505.99) | -0.23(-0.42 to -0.03) |
| Haiti | 421.87(345.68 to 505.39) | -0.11(-0.13 to -0.10) |
| Morocco | 419.39(341.86 to 502.36) | -0.33(-0.37 to -0.29) |
| New Zealand | 418.02(333.63 to 509.98) | -0.40(-0.59 to -0.21) |
| Tuvalu | 417.05(339.00 to 503.07) | 1.19(0.95 to 1.42) |
| Bosnia and Herzegovina | 415.34(340.29 to 493.31) | -1.02(-1.45 to -0.58) |
| Sudan | 415.33(342.53 to 498.81) | -0.34(-0.37 to -0.30) |
| Albania | 415.25(338.49 to 496.48) | -0.58(-0.63 to -0.54) |
| Netherlands | 414.14(336.09 to 497.46) | -0.42(-0.74 to -0.10) |
| Slovakia | 413.96(338.71 to 494.01) | -0.57(-0.62 to -0.52) |
| Kyrgyzstan | 412.99(338.45 to 492.99) | -0.25(-0.42 to -0.08) |
| Kazakhstan | 412.66(338.45 to 495.44) | -0.47(-0.54 to -0.39) |
| Philippines | 412.08(357.34 to 473.54) | 0.03(-0.03 to 0.10) |
| Palestine | 409.91(331.93 to 491.39) | -0.18(-0.20 to -0.17) |
| Syrian Arab Republic | 407.96(332.56 to 488.83) | -0.23(-0.27 to -0.20) |
| Serbia | 407.74(332.65 to 488.77) | -0.46(-0.52 to -0.39) |
| Montenegro | 405.59(333.58 to 483.21) | -0.45(-0.51 to -0.39) |
| Tokelau | 398.94(320.57 to 487.65) | -0.00(-0.23 to 0.23) |
| Hungary | 398.44(327.13 to 477.06) | -0.45(-0.59 to -0.31) |
| Nicaragua | 398.43(325.97 to 476.15) | -0.22(-0.24 to -0.20) |
| Honduras | 398.33(326.17 to 477.99) | -0.18(-0.18 to -0.17) |
| Guatemala | 396.25(325.74 to 475.19) | -0.21(-0.23 to -0.20) |
| Slovenia | 395.93(322.45 to 472.93) | -0.69(-0.93 to -0.44) |
| Georgia | 394.22(322.49 to 474.84) | -0.39(-0.57 to -0.20) |
| El Salvador | 392.29(320.23 to 469.36) | -0.24(-0.25 to -0.23) |
| Libya | 390.68(317.32 to 467.66) | 0.05(-0.03 to 0.14) |
| Dominican Republic | 389.83(317.23 to 468.44) | -0.46(-0.53 to -0.40) |
| Jordan | 389.10(316.42 to 468.37) | -0.31(-0.35 to -0.27) |
| Paraguay | 388.33(314.23 to 468.67) | -0.34(-0.66 to -0.01) |
| Armenia | 388.29(317.32 to 466.65) | -0.55(-0.71 to -0.40) |
| Czechia | 387.78(315.42 to 468.37) | -0.52(-0.73 to -0.31) |
| Nauru | 384.59(308.44 to 472.61) | 1.08(0.91 to 1.25) |
| Cuba | 383.74(310.68 to 467.88) | -0.32(-0.38 to -0.26) |
| Egypt | 378.52(307.00 to 455.33) | -0.36(-0.38 to -0.33) |
| Mongolia | 375.67(306.04 to 449.43) | -0.67(-0.81 to -0.53) |
| Iran (Islamic Republic of) | 375.18(305.06 to 458.56) | -0.15(-0.49 to 0.18) |
| Algeria | 374.76(303.77 to 454.05) | -0.22(-0.25 to -0.18) |
| Palau | 373.58(301.39 to 461.36) | 1.22(1.00 to 1.45) |
| Jamaica | 372.24(301.46 to 447.71) | -0.22(-0.24 to -0.21) |
| Belize | 371.83(301.54 to 450.87) | -0.26(-0.30 to -0.23) |
| Azerbaijan | 371.38(300.43 to 450.41) | -0.57(-0.79 to -0.36) |
| Lebanon | 370.95(302.25 to 444.43) | -0.33(-0.37 to -0.29) |
| Venezuela (Bolivarian Republic of) | 370.76(303.95 to 445.16) | -0.17(-0.21 to -0.13) |
| Tunisia | 370.10(299.51 to 447.88) | -0.36(-0.48 to -0.24) |
| Australia | 369.40(296.97 to 447.86) | -0.19(-0.60 to 0.22) |
| Guyana | 369.03(297.09 to 448.34) | -0.38(-0.39 to -0.38) |
| Iraq | 368.89(296.05 to 446.93) | -0.38(-0.45 to -0.32) |
| Bulgaria | 367.41(301.77 to 441.26) | -0.67(-0.90 to -0.45) |
| Ireland | 366.88(291.92 to 450.41) | -0.45(-0.75 to -0.15) |
| Mauritius | 365.51(294.43 to 440.25) | -0.40(-0.76 to -0.03) |
| Croatia | 365.10(297.93 to 445.44) | -0.81(-1.23 to -0.40) |
| Turkmenistan | 364.72(296.68 to 441.69) | -0.61(-0.79 to -0.43) |
| Costa Rica | 364.34(295.54 to 439.98) | -0.27(-0.28 to -0.26) |
| Republic of Moldova | 363.41(299.05 to 438.37) | -0.17(-0.26 to -0.07) |
| Dominica | 361.80(295.67 to 434.54) | -0.38(-0.40 to -0.35) |
| Saint Vincent and the Grenadines | 360.51(290.15 to 437.79) | -0.40(-0.43 to -0.37) |
| Suriname | 357.19(286.31 to 431.69) | -0.33(-0.36 to -0.30) |
| Saint Lucia | 356.61(285.22 to 441.18) | -0.36(-0.37 to -0.35) |
| Bahrain | 356.43(288.67 to 430.57) | -0.21(-0.24 to -0.18) |
| Malaysia | 354.82(284.02 to 428.76) | -0.95(-1.61 to -0.28) |
| Grenada | 354.55(287.09 to 429.12) | -0.45(-0.47 to -0.42) |
| Romania | 353.04(286.98 to 429.95) | -0.82(-1.10 to -0.55) |
| Barbados | 352.76(283.97 to 432.18) | -0.21(-0.25 to -0.17) |
| Oman | 352.12(283.53 to 430.62) | -0.35(-0.40 to -0.29) |
| Panama | 349.34(283.16 to 419.89) | -0.41(-0.43 to -0.38) |
| Belgium | 348.58(279.25 to 425.41) | -0.25(-0.28 to -0.23) |
| Saudi Arabia | 348.09(281.43 to 423.52) | -0.26(-0.29 to -0.23) |
| Greece | 347.13(274.91 to 429.05) | -0.45(-0.77 to -0.13) |
| Colombia | 345.58(280.31 to 421.31) | -0.29(-0.32 to -0.27) |
| Antigua and Barbuda | 343.63(275.32 to 418.89) | -0.29(-0.31 to -0.28) |
| Norway | 342.70(292.01 to 400.19) | -0.74(-1.57 to 0.10) |
| Kuwait | 340.70(272.64 to 418.50) | -0.35(-0.39 to -0.31) |
| United States of America | 339.50(279.25 to 409.14) | -0.75(-1.18 to -0.31) |
| Austria | 338.46(270.15 to 417.27) | -0.40(-0.64 to -0.17) |
| Uzbekistan | 338.41(274.68 to 406.61) | -0.51(-0.69 to -0.33) |
| Bahamas | 337.47(272.49 to 414.31) | -0.20(-0.24 to -0.17) |
| Saint Kitts and Nevis | 336.03(268.80 to 410.62) | -0.42(-0.45 to -0.39) |
| Puerto Rico | 335.72(270.55 to 412.13) | -0.47(-0.86 to -0.07) |
| Belarus | 332.89(271.27 to 404.07) | -0.31(-0.39 to -0.23) |
| Finland | 331.89(264.92 to 410.70) | -1.52(-2.03 to -1.01) |
| United Kingdom | 326.48(273.26 to 387.54) | -0.45(-0.86 to -0.03) |
| Trinidad and Tobago | 326.24(260.97 to 403.63) | -0.60(-0.64 to -0.56) |
| Estonia | 322.79(262.89 to 392.08) | -0.68(-0.97 to -0.38) |
| Solomon Islands | 320.57(258.13 to 391.18) | -0.09(-0.11 to -0.06) |
| Kiribati | 320.03(256.87 to 389.42) | -0.06(-0.07 to -0.04) |
| Bermuda | 315.97(253.75 to 385.60) | -0.38(-0.41 to -0.34) |
| South Africa | 314.44(255.94 to 369.57) | -0.59(-0.96 to -0.22) |
| Vanuatu | 314.32(254.17 to 382.27) | -0.10(-0.11 to -0.09) |
| Cyprus | 313.70(250.04 to 389.67) | -0.28(-0.32 to -0.23) |
| Marshall Islands | 313.33(250.31 to 383.38) | -0.07(-0.08 to -0.05) |
| Israel | 312.63(247.78 to 385.13) | -0.28(-0.42 to -0.14) |
| Argentina | 310.84(246.24 to 383.00) | -0.38(-0.41 to -0.35) |
| Portugal | 310.26(246.14 to 379.91) | -0.28(-0.32 to -0.23) |
| Papua New Guinea | 310.03(249.85 to 376.67) | -0.12(-0.14 to -0.09) |
| Micronesia (Federated States of) | 309.39(247.05 to 378.57) | -0.11(-0.12 to -0.11) |
| Uruguay | 306.96(242.97 to 377.24) | -0.42(-0.49 to -0.35) |
| Chile | 306.94(242.85 to 378.15) | -0.53(-0.57 to -0.50) |
| Luxembourg | 306.27(243.20 to 382.42) | -0.38(-0.58 to -0.17) |
| Indonesia | 305.75(260.88 to 362.66) | -0.10(-0.16 to -0.04) |
| Democratic People's Republic of Korea | 302.50(242.08 to 370.52) | 0.46(0.44 to 0.48) |
| Iceland | 301.49(238.24 to 375.31) | -0.25(-0.27 to -0.22) |
| San Marino | 299.80(238.54 to 373.24) | -0.12(-0.19 to -0.05) |
| Samoa | 298.69(236.86 to 364.88) | -0.21(-0.22 to -0.20) |
| Namibia | 297.25(244.41 to 348.39) | -0.33(-0.42 to -0.24) |
| Italy | 297.21(236.50 to 367.35) | -0.31(-0.67 to 0.04) |
| Tonga | 297.19(237.23 to 369.50) | -0.19(-0.20 to -0.18) |
| Germany | 296.27(232.56 to 365.93) | -0.87(-1.39 to -0.35) |
| Cambodia | 295.45(237.02 to 363.22) | -0.52(-0.56 to -0.48) |
| Zimbabwe | 292.91(243.00 to 346.06) | 0.11(-0.04 to 0.27) |
| Latvia | 292.79(238.05 to 358.51) | -0.89(-1.34 to -0.44) |
| Denmark | 290.59(229.77 to 359.39) | -0.75(-1.41 to -0.09) |
| China | 288.72(237.41 to 347.79) | -0.13(-0.41 to 0.15) |
| Sweden | 288.68(215.14 to 376.92) | 0.54(0.12 to 0.96) |
| Andorra | 288.61(226.49 to 358.90) | -0.16(-0.20 to -0.12) |
| Lesotho | 287.99(239.36 to 339.77) | -0.31(-0.33 to -0.29) |
| Fiji | 284.72(223.23 to 353.90) | -0.20(-0.22 to -0.18) |
| Lithuania | 280.16(227.41 to 344.90) | -0.79(-1.10 to -0.47) |
| Timor-Leste | 279.02(224.13 to 344.34) | -0.49(-0.54 to -0.43) |
| Niue | 278.77(219.11 to 348.65) | -0.26(-0.28 to -0.24) |
| American Samoa | 278.75(222.78 to 348.92) | 0.03(0.02 to 0.04) |
| France | 275.35(218.52 to 344.41) | -0.16(-0.60 to 0.27) |
| Switzerland | 275.29(217.81 to 337.09) | -0.59(-0.82 to -0.35) |
| United States Virgin Islands | 274.97(219.33 to 338.23) | -0.68(-1.38 to 0.02) |
| Monaco | 263.89(208.43 to 332.78) | -0.15(-0.21 to -0.10) |
| Spain | 262.76(207.63 to 325.14) | -1.08(-1.64 to -0.52) |
| Northern Mariana Islands | 259.98(206.72 to 329.06) | 0.11(0.07 to 0.14) |
| Thailand | 259.57(206.27 to 321.43) | -0.48(-0.50 to -0.46) |
| Cook Islands | 258.24(201.68 to 322.94) | -0.29(-0.31 to -0.28) |
| India | 249.33(211.72 to 290.12) | 0.07(-0.62 to 0.76) |
| Maldives | 248.86(196.76 to 314.68) | -0.56(-0.59 to -0.53) |
| Botswana | 248.79(203.08 to 295.63) | -0.36(-0.37 to -0.34) |
| Malta | 247.49(195.44 to 308.03) | -0.42(-0.45 to -0.39) |
| Seychelles | 246.57(196.20 to 307.91) | -0.37(-0.41 to -0.34) |
| Guam | 244.46(190.53 to 305.72) | -0.11(-0.16 to -0.07) |
| Viet Nam | 243.38(192.24 to 302.21) | -0.66(-0.99 to -0.32) |
| Central African Republic | 236.14(191.30 to 280.58) | -0.05(-0.08 to -0.02) |
| Sri Lanka | 235.37(185.94 to 293.07) | -1.02(-1.63 to -0.42) |
| Democratic Republic of the Congo | 234.36(189.86 to 277.06) | 0.01(-0.10 to 0.12) |
| Pakistan | 232.72(188.50 to 283.73) | -0.37(-0.79 to 0.06) |
| Japan | 232.28(191.09 to 279.89) | -1.42(-2.12 to -0.72) |
| Taiwan (Province of China) | 230.53(180.11 to 290.09) | 0.18(0.15 to 0.21) |
| Greenland | 230.49(185.55 to 283.97) | -0.28(-0.30 to -0.26) |
| Qatar | 228.12(180.11 to 279.91) | -0.54(-0.73 to -0.35) |
| Eswatini | 218.18(177.42 to 259.14) | -0.24(-0.34 to -0.14) |
| Coted'Ivoire | 202.91(163.25 to 247.62) | -0.26(-0.33 to -0.19) |
| Senegal | 202.35(165.02 to 244.26) | -0.30(-0.37 to -0.24) |
| Lao People's Democratic Republic | 200.18(158.15 to 251.71) | -0.69(-0.98 to -0.41) |
| Mauritania | 199.50(160.25 to 242.41) | -0.38(-0.56 to -0.19) |
| Chad | 199.24(161.18 to 241.76) | -0.50(-0.68 to -0.32) |
| Congo | 197.78(161.23 to 236.62) | -0.30(-0.37 to -0.23) |
| Angola | 194.04(158.50 to 232.66) | -0.49(-0.56 to -0.41) |
| Myanmar | 192.08(151.35 to 240.57) | -0.76(-1.16 to -0.36) |
| Canada | 188.63(147.84 to 232.88) | -0.02(-0.45 to 0.40) |
| Niger | 182.88(146.15 to 223.58) | -0.23(-0.31 to -0.16) |
| Liberia | 182.01(146.16 to 224.20) | -0.28(-0.39 to -0.18) |
| Gabon | 179.02(145.78 to 216.08) | -0.19(-0.22 to -0.16) |
| Brunei Darussalam | 178.35(138.93 to 223.08) | 0.01(-0.00 to 0.01) |
| Sierra Leone | 175.86(141.58 to 213.70) | -0.24(-0.33 to -0.14) |
| Guinea-Bissau | 174.82(139.85 to 216.63) | -0.24(-0.29 to -0.19) |
| Togo | 174.78(140.23 to 211.74) | -0.25(-0.32 to -0.18) |
| Singapore | 174.68(136.24 to 221.52) | -0.39(-0.40 to -0.38) |
| Equatorial Guinea | 172.56(140.71 to 208.07) | -1.22(-1.39 to -1.05) |
| Republic of Korea | 172.07(134.95 to 214.76) | -0.22(-0.84 to 0.40) |
| Gambia | 169.98(136.12 to 208.51) | -0.19(-0.25 to -0.14) |
| Guinea | 169.89(135.88 to 209.49) | -0.26(-0.33 to -0.19) |
| Benin | 168.61(136.09 to 204.51) | -0.39(-0.46 to -0.33) |
| Sao Tome and Principe | 164.23(130.82 to 200.79) | -0.43(-0.51 to -0.35) |
| Cameroon | 163.69(131.43 to 200.94) | -0.30(-0.38 to -0.21) |
| Somalia | 163.46(131.80 to 197.80) | 0.12(0.07 to 0.17) |
| Ghana | 158.84(127.59 to 196.05) | -0.52(-0.68 to -0.36) |
| Cabo Verde | 154.76(124.82 to 192.85) | -0.65(-0.71 to -0.59) |
| Nigeria | 145.78(123.71 to 173.59) | -0.80(-1.08 to -0.52) |
| Bhutan | 145.50(115.26 to 181.34) | -0.82(-0.84 to -0.80) |
| Comoros | 142.06(115.45 to 174.32) | -0.05(-0.09 to -0.01) |
| Kenya | 139.59(112.24 to 171.73) | -0.17(-0.26 to -0.08) |
| Burundi | 139.43(112.36 to 170.32) | 0.02(-0.01 to 0.05) |
| Malawi | 138.75(111.56 to 169.39) | -0.14(-0.21 to -0.07) |
| Mali | 135.55(108.97 to 165.75) | -0.47(-0.67 to -0.26) |
| Mozambique | 134.71(109.31 to 165.47) | -0.37(-0.40 to -0.34) |
| Ethiopia | 133.01(108.22 to 161.89) | -0.30(-0.46 to -0.14) |
| Eritrea | 130.44(104.97 to 159.25) | -0.15(-0.18 to -0.11) |
| Madagascar | 130.13(104.45 to 160.09) | -0.05(-0.08 to -0.02) |
| Uganda | 128.90(103.92 to 159.17) | -0.29(-0.31 to -0.26) |
| Rwanda | 128.77(103.31 to 158.27) | -0.29(-0.36 to -0.22) |
| South Sudan | 127.25(102.29 to 156.43) | 0.18(0.12 to 0.24) |
| Burkina Faso | 124.49(98.98 to 151.91) | -0.56(-0.71 to -0.41) |
| United Republic of Tanzania | 123.98(99.00 to 151.95) | -0.28(-0.33 to -0.23) |
| Zambia | 120.57(96.12 to 148.24) | -0.28(-0.40 to -0.16) |
| Djibouti | 119.05(95.37 to 148.24) | -0.09(-0.14 to -0.04) |
| Nepal | 114.03(91.00 to 142.80) | -0.90(-0.92 to -0.88) |
| Bangladesh | 106.34(83.92 to 133.25) | -0.82(-0.86 to -0.78) |

**Table S3** Results of the Weighted Restricted Cubic Spline Model for the Association Between SDI and ASIR Across 204 Countries

| Parameter | Estimate | SE | t-value | *p-value* |
| --- | --- | --- | --- | --- |
| Intercept | 120.21 | 31.15 | 3.86 | <0.001 |
| SDI (Linear) | 106.66 | 75.63 | 1.41 | 0.16 |
| SDI (Second-order) | 1,061.95 | 193.79 | 5.48 | <0.001 |
| SDI (Third-order) | 9,012.23 | 1,358.65 | -6.63 | <0.001 |
| **Model Fit Statistics** |  |  |  |  |
| R² | 0.49 | — | — | — |
| Adjusted R² | 0.48 | — | — | — |
| Residual standard error (σ) | 2.91 | — | — | — |
| F statistic (SDI overall) | — | — | 63.48 | <0.001 |
| Wald test for non-linearity (df=2) | — | — | 24.49 | <0.001 |

**Table S4** Results from random-effects meta-regression of ASR on SDI

| Variable | Estimate (95% CI) | SE | z-value | p-value |
| --- | --- | --- | --- | --- |
| Intercept | 41.84 (-50.22, 133.90) | 46.97 | 0.89 | 0.37 |
| SDI_spline1 | 419.26 (206.25, 632.27) | 108.68 | 3.86 | <0.001 |
| SDI_spline2 | 227.10 (-237.54, 691.73) | 237.06 | 0.96 | 0.34 |
| SDI_spline3 | -4,331.84 (-7,240.17, -1,423.51) | 1,483.87 | -2.92 | 0.004 |
| **Model Fit** |  |  |  |  |
| τ² | 6,092.40 |  |  |  |
| I² | 90.22% |  |  |  |

**Table S5 Joinpoint regression analysis: trends in age-standardized incidence rates (per 100,000 population) among males, and females global, 1990–2021**

| Gender | Period | Annual Percent Change (95% CI) | AAPC (95% CI) |
| --- | --- | --- | --- |
| Male | 1990-1994 | -1.30 (-1.55 to -1.05) | -0.24 (-0.34 to -0.15) |
|  | 1994-2007 | 0.05 (0.00 to 0.09) |  |
|  | 2007-2010 | 0.72 (0.06 to 1.40) |  |
|  | 2010-2015 | -2.50 (-2.70 to -2.30) |  |
|  | 2015-2019 | 1.68 (1.33 to 2.03) |  |
|  | 2019-2021 | 0.45 (-0.22 to 1.13) |  |
| Female | 1990-2020 | -1.02 (-1.08 to -0.96) | -0.24 (-0.31 to -0.17) |
|  | 2020-2010 | 0.69 (0.63 to 0.75) |  |
|  | 2010-2015 | -2.19 (-2.38 to -1.99) |  |
|  | 2015-2019 | 1.64 (1.31 to 1.98) |  |
|  | 2019-2021 | 0.29 (-0.35 to 0.94) |  |

AAPC: average annual percent change presented for full period; CI: confidence interval.

**Table S6 Out-of-Sample Prediction Errors of Nordpred and BAPC Models Across Sex Groups**

| Model | Sex Group | MSE | MAE | MAPE (%) |
| --- | --- | --- | --- | --- |
| Nordpred | Both | 807.64 | 27.56 | 9.34 |
| Nordpred | Female | 876.65 | 28.76 | 9.14 |
| Nordpred | Male | 737.46 | 26.27 | 9.54 |
| BAPC | Both | 170.19 | 11.16 | 3.82 |
| BAPC | Female | 101.84 | 7.85 | 2.53 |
| BAPC | Male | 250.73 | 14.1 | 5.23 |

MSE: Mean Squared Error; MAE: Mean Absolute Error; MAPE: Mean Absolute Percentage Error.
