# Supplementary material for: The global, regional, and national patterns of change in the burden of edentulism, 1990–2021: an analysis of the global burden of disease study 2021 and forecast to 2041
Source: Front Oral Health. 2025 Dec 1;6:1678201. doi: 10.3389/froh.2025.1678201 (PMC12702965; doi:10.3389/froh.2025.1678201)
Supplement: Supplementary file 2 [file Table2.docx]

**
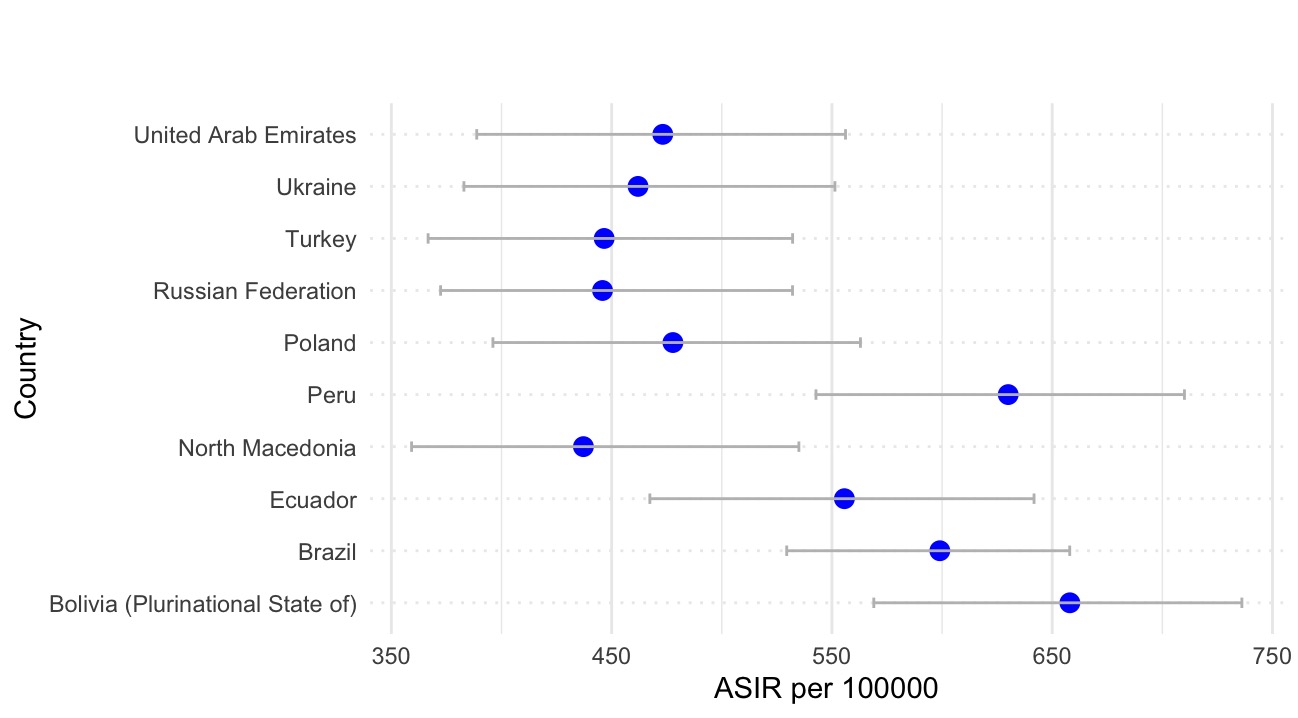
**

**Figure S1** Age-standardized incidence rate (ASIR) of edentulism in the top 10 countries in 2021.


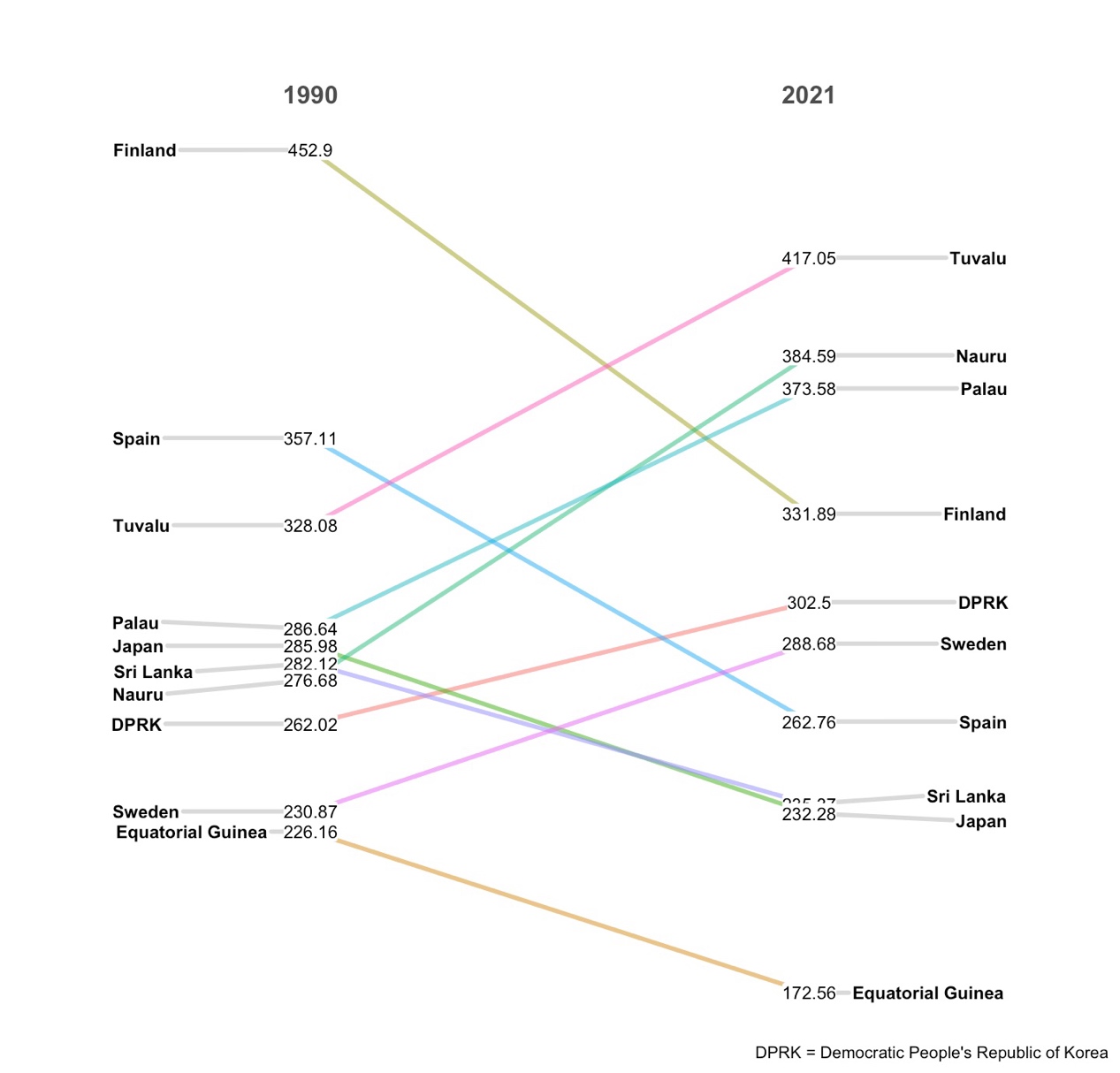


**Figure S2** Slopegraph showing the age-standardized incidence rate (ASIR) trends of edentulism in countries with the top 5 and bottom 5 estimated annual percentage changes (EAPC) from 1990 to 2021.


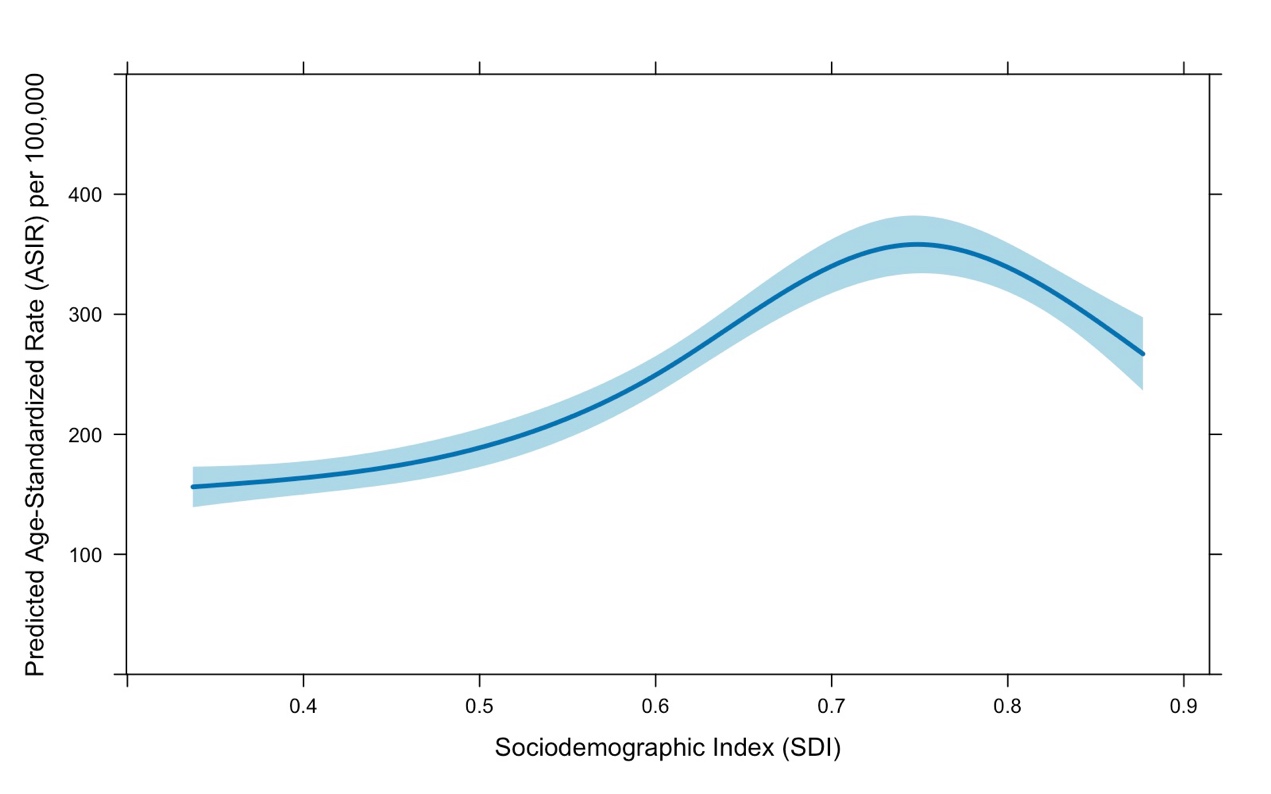


**Figure S3** Non-linear association between Sociodemographic Index (SDI) and age-standardized incidence rate (ASIR) .
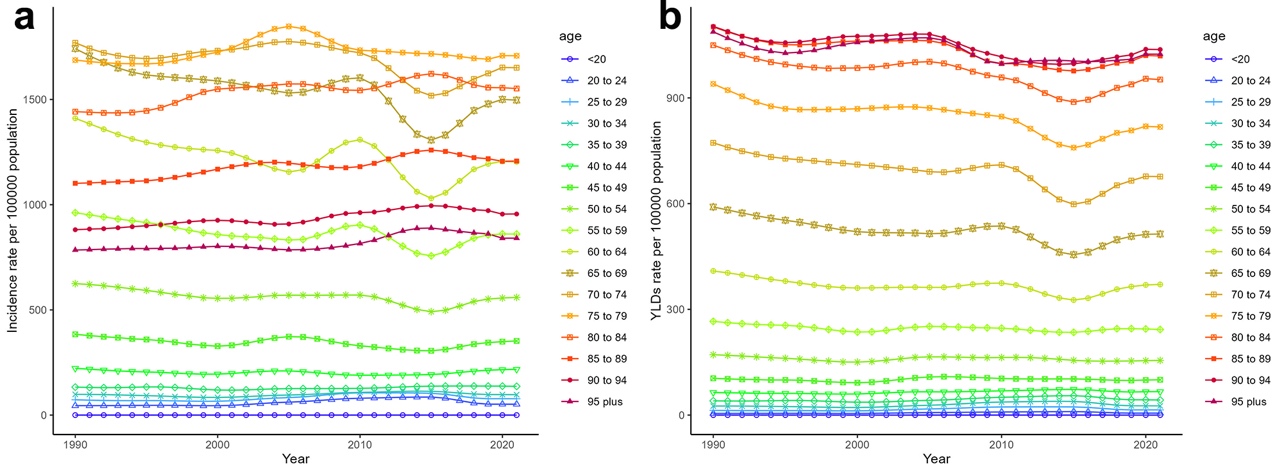


**Figure S4.** Time trends of the incidence rate (a) and YLDs (b) of edentulism across different 17 age groups from 1990 to 2021.
