# Supplementary figures and images for: The global, regional, and national patterns of change in the burden of edentulism, 1990–2021: an analysis of the global burden of disease study 2021 and forecast to 2041
Source: Front Oral Health. 2025 Dec 1;6:1678201. doi: 10.3389/froh.2025.1678201 (PMC12702965; doi:10.3389/froh.2025.1678201)

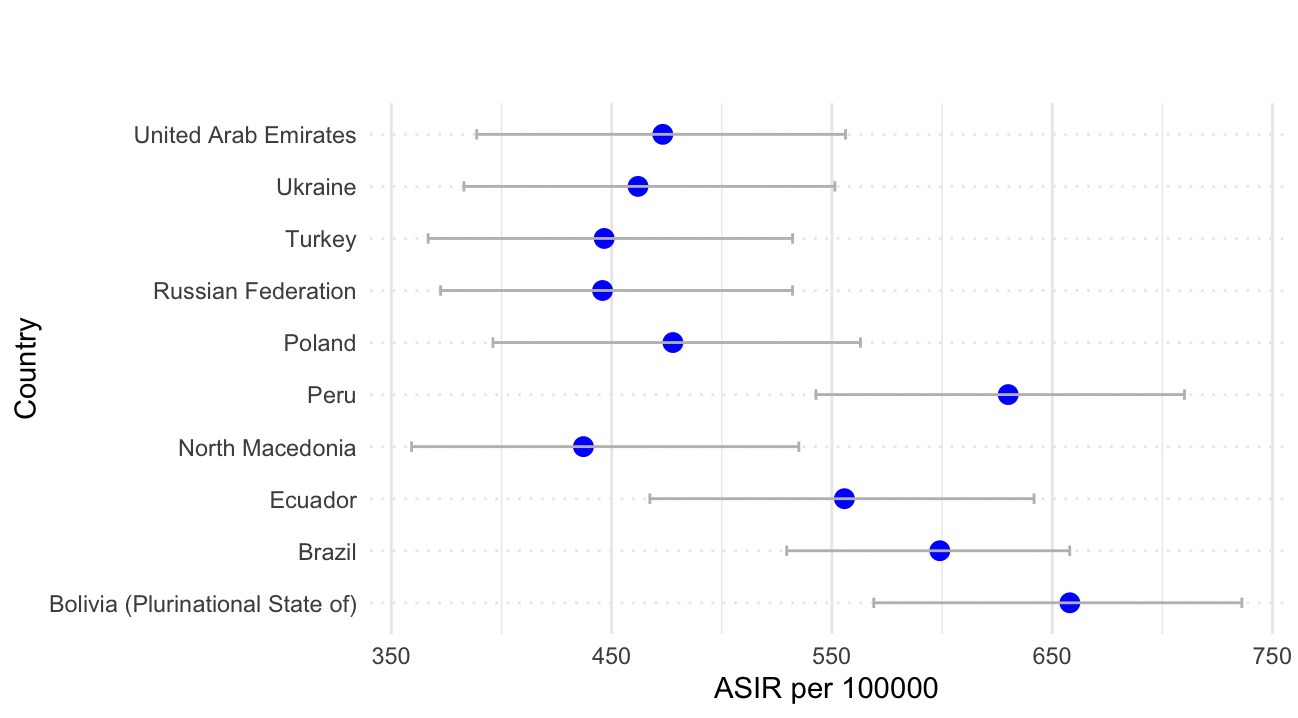

Supplement: Supplementary file 4 [file Image1.jpeg]

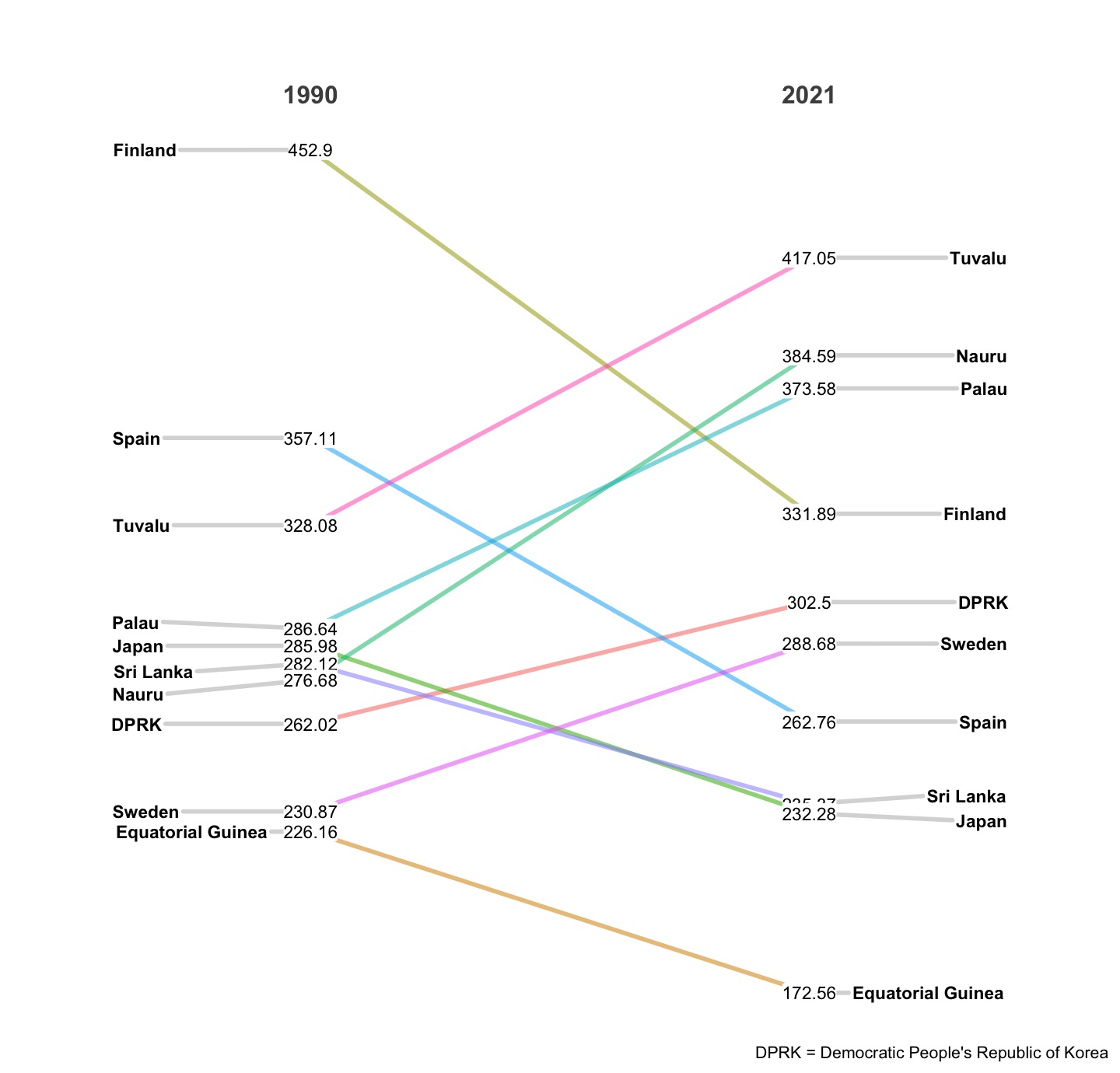

Supplement: Supplementary file 5 [file Image2.jpeg]

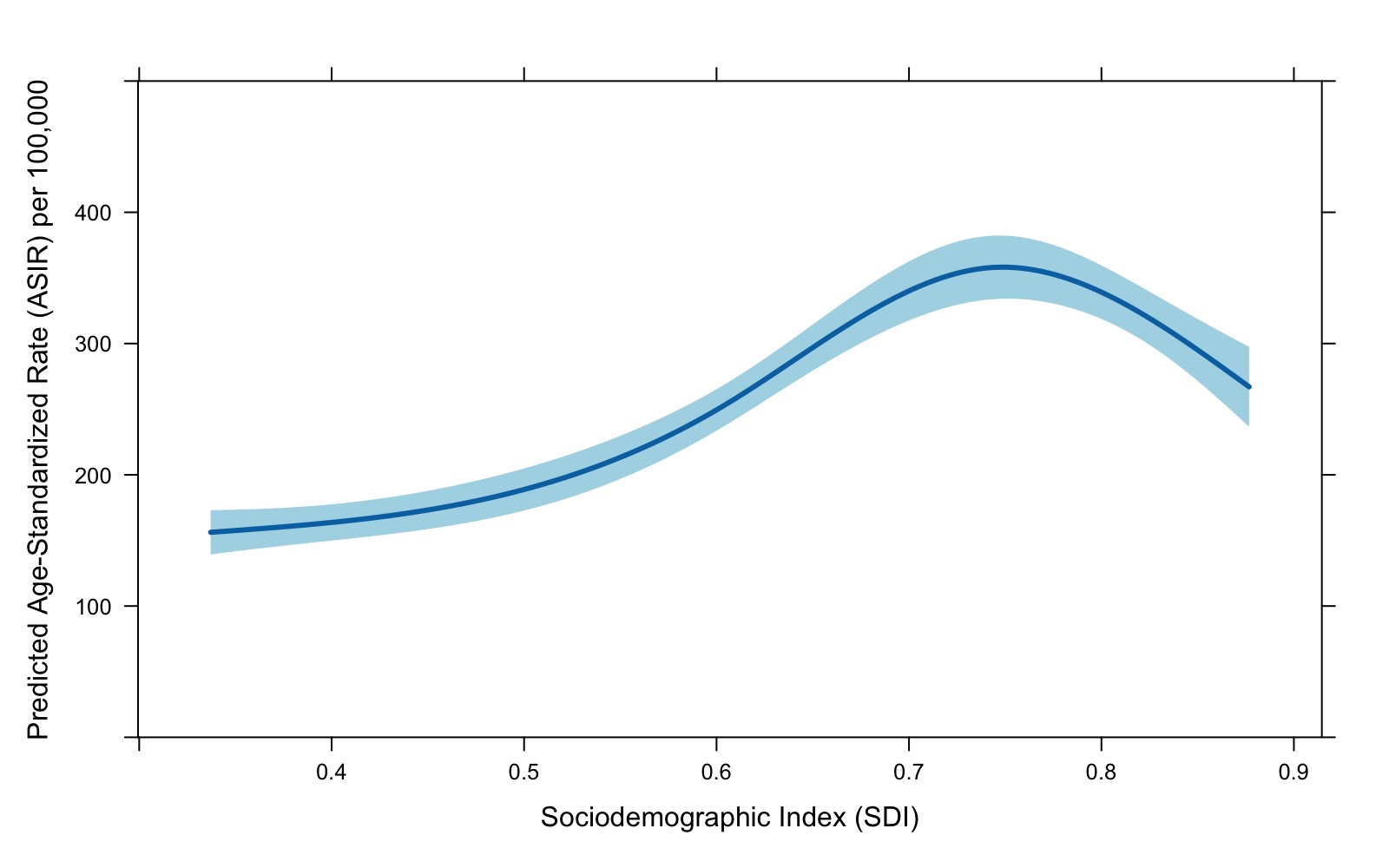

Supplement: Supplementary file 6 [file Image3.jpeg]

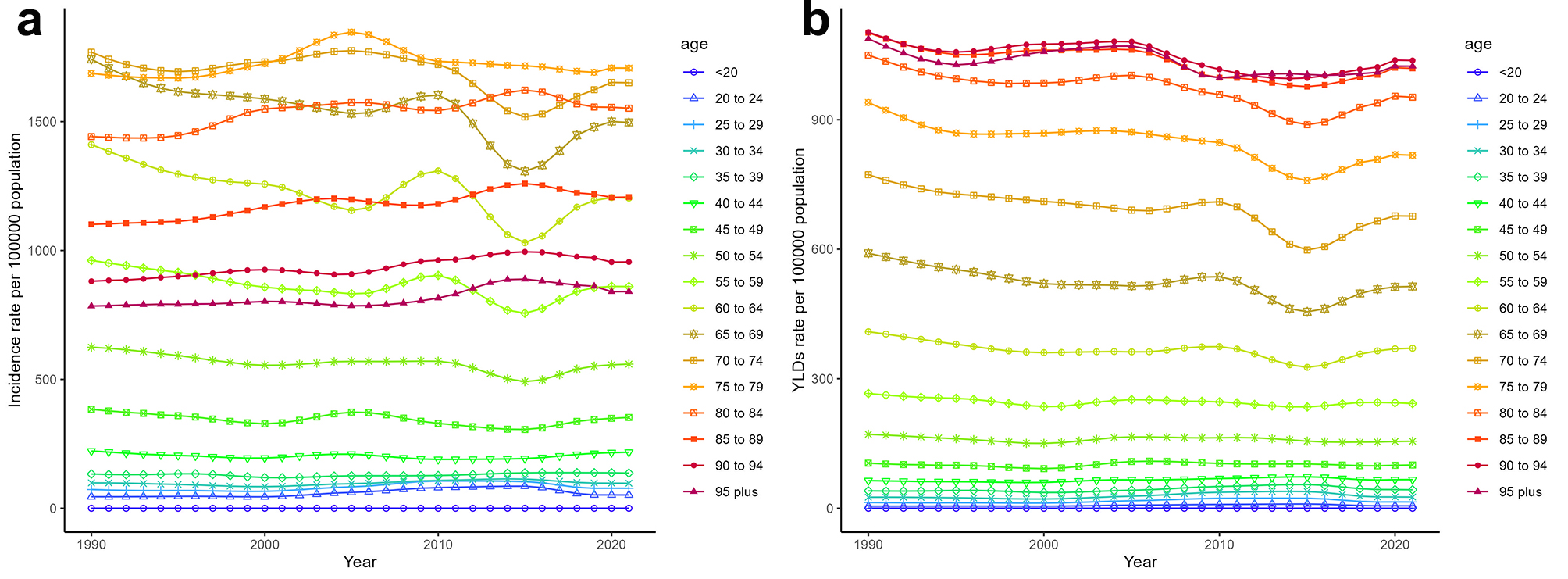

Supplement: Supplementary file 7 [file Image4.jpeg]
